# Creation and control of high-dimensional multi-partite classically entangled light

**DOI:** 10.1038/s41377-021-00493-x

**Published:** 2021-03-08

**Authors:** Yijie Shen, Isaac Nape, Xilin Yang, Xing Fu, Mali Gong, Darryl Naidoo, Andrew Forbes

**Affiliations:** 1 School of Physics, University of the Witwatersrand, Private Bag 3, Wits, 2050 South Africa; 2 State Key Laboratory of Precision Measurement Technology and Instruments, Department of Precision Instrument, Tsinghua University, 100084 Beijing, China; 3 Electrical and Computer Engineering Department, University of California, Los Angeles, CA 90095 USA; 4 Key Laboratory of Photonic Control Technology (Tsinghua University), Ministry of Education, 100084 Beijing, China; 5 CSIR National Laser Centre, PO Box 395, Pretoria, 0001 South Africa; 6 Present Address: Optoelectronics Research Centre, University of Southampton, Southampton, SO17 1BJ UK

**Keywords:** Solid-state lasers, Quantum optics

## Abstract

Vector beams, non-separable in spatial mode and polarisation, have emerged as enabling tools in many diverse applications, from communication to imaging. This applicability has been achieved by sophisticated laser designs controlling the spin and orbital angular momentum, but so far is restricted to only two-dimensional states. Here we demonstrate the first vectorially structured light created and fully controlled in eight dimensions, a new state-of-the-art. We externally modulate our beam to control, for the first time, the complete set of classical Greenberger–Horne–Zeilinger (GHZ) states in paraxial structured light beams, in analogy with high-dimensional multi-partite quantum entangled states, and introduce a new tomography method to verify their fidelity. Our complete theoretical framework reveals a rich parameter space for further extending the dimensionality and degrees of freedom, opening new pathways for vectorially structured light in the classical and quantum regimes.

## Introduction

In recent years, structured light, the ability to arbitrarily tailor light in its various degrees of freedom (DoFs), has risen in prominence^1–3^, particularly the vectorially structured light^4–6^, which is non-separable in spatial mode and polarisation. A popular example is vector vortex beams, a vectorial combination of spin and orbital angular momentum (OAM) states, as a form of a two-dimensional classically entangled state^7–10^. Sharing the same hallmark of non-separability of quantum entanglement, the classically entangled vector beam is more than simple mathematical machinery and can extend a myriad of applications with quantum-classical connection. Such states of vectorially structured light have been created external to the source through interferometric approaches by spin–orbit optics^11–14^, as well as by customised lasers^15^ including custom fibre lasers^16^, intra-cavity geometric phase elements in solid-state lasers^17–20^ and custom on-chip solutions^21–23^. The resulting beams have proved instrumental in imaging^24^, optical trapping and tweezing^25,26^, metrology^27–29^, communication^30,31^ and simulating quantum processes^8–10,32–36^. In the quantum regime, they are referred to as hybrid entangled states and have likewise been used extensively in quantum information processing and cryptography^37–39^.

Despite these impressive advances, the prevailing paradigm is limited in two-DoF (bipartite) and two-dimensional (2D) classically entangled states of light, the classical analogy to two-photon qubit entanglement, which has proved useful in describing such beams as states on a sphere^40,41^. The ability to access more DoFs and arbitrarily engineered high-dimensional state spaces with vectorial light would be highly beneficial, opening the way to many exciting applications in simulating multi-partite quantum processes in simpler laboratory settings^42,43^, in advancing our understanding of spin–orbit coupling through new coupling paradigms between spin and the trajectory of light^44,45^, and in accessing more DoFs and dimensions in the single-photon and coherent states for high-capacity communication^46–49^. To do so requires the creation and control of new DoFs in vectorially structured light.

Existing vectorial control is very powerful^6^ but does not easily extend the DoFs. One could carry out the spatial manipulation of light to partition the spatial DoFs, e.g., the scalar modes into their two indices (*n* and *m* for the Hermite-Gaussian modes, *p* and *ℓ* for the Laguerre-Gaussian modes), but the DoFs remain limited to three^39,50^, and independent control is practically impossible with the present tools, e.g., how can one change the phase of only the radial modes and not the azimuthal modes in the Laguerre-Gaussian basis? It is possible to extend the DoFs by the time-frequency or wavelength control of light^51–53^, but this is non-trivial and involves nonlinear materials. One could split the beam into multiple paths^54–61^ but then the DoFs would no longer be intrinsic to one paraxial beam and control would become increasingly complicated and problematic. A recent work extended the DoFs up to three but still limited to 2D states^62^, that cannot be fully controlled in high-dimensional space. The open challenge is therefore to find unlimited DoFs that are easy to control, intrinsic to a paraxial beam, and have the potential to access high dimensions with classical light, a topic very much in its infancy.

**Fig. 1:**
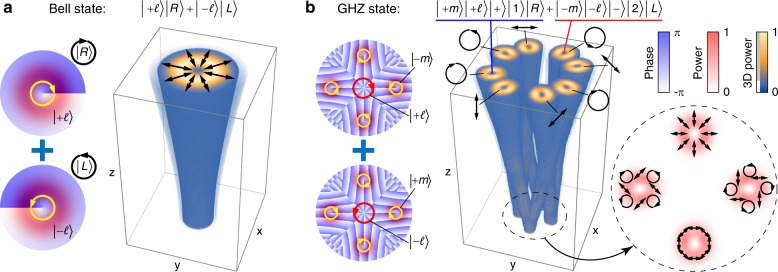
Vectorially structured light. **a** A conventional vector beam: a paraxial mode with a spatially varying polarisation structure, characterised by the given Bell state, and **b** an illustration of a new high-dimensional vectorially structured light field comprising polarisation marked lights along multiple intrinsic DoFs in one single paraxial beam, which is constructed by a set of GHZ states. The Bell state and GHZ state are marked in **a** and **b**, respectively. *x* and *y* are the transverse coordinates and *z* is the longitudinal coordinate (paraxial propagation direction)

Here, we introduce the notion that the intrinsic DoFs from a ray-wave duality laser can be marked and controlled for high-dimensional multi-partite (multi-DoF) classically entangled states of vectorial light. We operate a laser in a frequency-degenerate state that is known to produce multiple ray-like trajectories but in a single wave-like paraxial beam as a spatial wave packet of SU(2) coherent state^63–66^ and vectorise it by using off-axis pumping and an anisotropic crystal^62,67,68^. However, the ray states in this beam cannot be tuned independently and therefore increase the difficulty of the arbitrary control of these high-dimensional states. We propose the combination of ray-wave duality in a laser beam and external digital modulation to overcome this paradigm. This method allows us to produce new forms of vectorial structured light not observed before, in stark contrast to conventional vector beams expressed by space-polarisation non-separable Bell states (Fig. 1a). As we will show, the ray trajectory and vectorial control for a single coherent paraxial beam gives us access to new controllable DoFs to realise high-dimensional classical entanglement. Specifically, we can use the intrinsic DoFs such as the oscillating direction ($\left|\pm \right\rangle$), ray location ($\left|1\right\rangle$ or $\left|2\right\rangle$), polarisation ($\left|R\right\rangle$ or $\left|L\right\rangle$ for right- or left-handed circular polarisation) and OAM and sub-OAM ($\left|\pm \!\ell \right\rangle$ and $\left|\pm\! m\right\rangle$) to realise multi-partite high-dimensional classically entangled light from a laser (Fig. 1b). As illustrated in Fig. 1, one can view conventional vector beams (Fig. 1a) as a subspace within this more general classically entangled state of vectorial structured light (Fig. 1b).

To demonstrate that these DoFs are fully controllable in a high-dimensional multi-partite space, we experimentally exploit a ray-wave structured laser beam in a tri-partite eight-dimensional state and controllably modulate it to produce, for the first time, the complete set of classical Greenberger–Horne–Zeilinger (GHZ) states in vector beams, the maximally entangled states in an eight-dimensional Hilbert space?, as the classical analogy to multi-partite or multi-particle entanglement. Our work introduces new DoFs beyond those of the traditional vector beams and creates new states of non-separable structured light not realised before.

## Results

### Concept

Spatial light mode control in lasers allows one to specify the desired modal amplitude, phase and polarisation^15^. While the spatial modes usually refer to the eigenmodes of the paraxial wave equation, there is also a class of complex spatial wave-packet modes that possess a geometric interpretation with SU(2) symmetry, a general symmetry for describing paraxial structured beams with OAM evolution mapped on a Poincaré-like sphere^69^. This geometric mode has the formation of SU(2) coherent state, with the salient property that the distribution of the wave function is coupled with a classical movement trajectory^64,70^. This means that the spatial wave pattern can also be treated as a cluster of geometric rays, a form of ray-wave duality^65,71^, a notion we will shortly exploit.

In this paper, we wish to demonstrate an eight-dimensional state from the laser in order to go on and create the GHZ states. To do this we build a ray-wave duality laser resonator as our source and select the new DoFs to be the oscillating direction, round-trip location, and polarisation. Such ray-wave duality light can be generated from an off-axis pumped laser cavity with frequency degeneracy, where the ratio of the transverse and longitudinal mode frequency spacings (Δ*f*_T_ and Δ*f*_L_) is a rational number Ω = Δ*f*_T_/Δ*f*_L_ = *P*/*Q* (*P* and *Q* are coprime), related to the period of the ray trajectory oscillating in the cavity (*Q* determines the number of rays)^64,65^. To this end, we use the degenerate state of $\left|{{\Omega }}=1/4\right\rangle$ to illustrate our method. This is achieved when the cavity length is precisely controlled as the half of the radius of curvature of the concave mirror in a plano-concave cavity, and the ray trajectory has a period of four round-trips in the cavity (see more general cases in Supplementary Information A). Then we carefully apply off-axis pumping to subsequently excite a W-shaped trajectory mode whose output mode can be shown to be exactly the desired SU(2) coherent state^72^. Although ray-like, the output is a paraxial beam with a coherent spatial wave packet based on the ray-wave duality, enabling us to label the output in terms of the ray trajectories rather than the modes themselves.

**Fig. 2:**
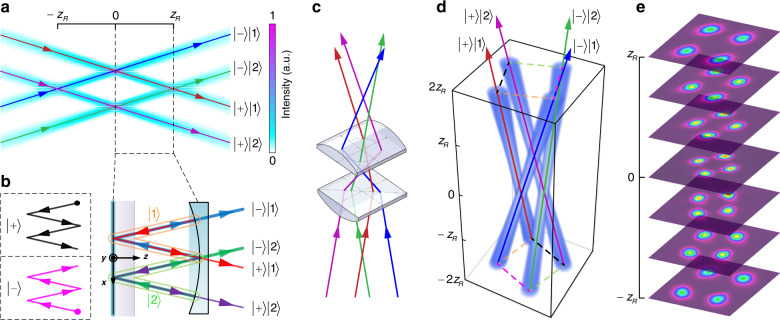
Laser concept. **a** A 2D planar representation of the desired geometric mode, where the mode evolves from wave-like fringes (at *z* = 0 and ±*z*_R_) to a ray-like trajectory. The means to create this mode in the cavity is illustrated in **b**, where completed oscillation orbits can be described by the direction states $\left|+\right\rangle$ and $\left|-\right\rangle$ (equivalently the sub-orbits shown in black and pink, respectively, in the dashed-line boxes), as well as paths originating from ray positions $\left|1\right\rangle$ and $\left|2\right\rangle$ (equivalently the sub-orbits shown in orange and green, respectively). **c** The planar mode is converted to the skewed version with a pair of cylindrical lenses. **d** A 3D schematic of a skewed (vortex) SU(2) geometric mode propagating in free space, with **e** experimental beam images shown at example propagation distances

**Fig. 3:**
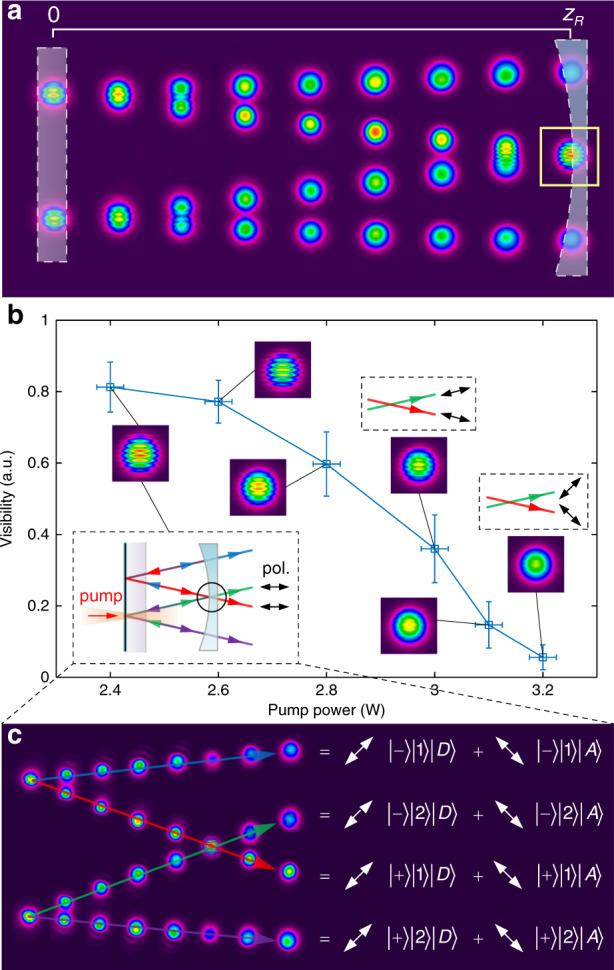
Vectorial ray-wave beams. **a** Experimental verification of an SU(2) geometric mode exhibiting ray-wave duality, where the corresponding positions of the flat and concave cavity mirrors are shown. The images show the evolution of the output mode from one end of the cavity to the other as a sequence of camera images. When the rays overlap, the wave-like nature is evident, as seen by the fringes shown in the yellow box. **b** The polarisation structure of the rays can be adjusted by tuning the pumping, inducing a change in fringe visibility with the pump power as the polarisation state of each ray is modified. The insets show the measured interference fringes at *z* = *z*_R_ for selected pump powers. The dashed boxes depict the corresponding ray representation together with the polarisation change. **c** The notation of all the elements in eight dimensions in an output vectorial ray-wave beam

A free-space planar ray-wave geometric mode with the highlighted ray trajectory of $\left|{{\Omega }}=1/4\right\rangle$ is shown in Fig. 2a, and the corresponding oscillation in the laser cavity is shown in Fig. 2b, with the forward ($\left|+\right\rangle$) and backward ($\left|-\right\rangle$) oscillating states and the first ($\left|1\right\rangle$) and second ($\left|2\right\rangle$) round-trip location states forming a completed oscillation with the potential to reach dimension *d* = 4. In Fig. 2a, the ray-like propagation is revealed by the marked trajectories, and at *z* = 0 and ±*z*_R_ (*z*_R_ is the Rayleigh range), the wave-like behaviour is evident by the fringes in the beam as trajectories overlap. To understand how to create this from a laser, we highlight the generation step inside a laser in Fig. 2b. Here, the output four rays originating from two locations ($\left|1\right\rangle ,\left|2\right\rangle$) on the rear mirror comprise two V-shaped locations, and the pair of rays in a certain location state have two different directions ($\left|+\right\rangle ,\left|-\right\rangle$), such that the output is spanned by the basis states ${{\mathcal{H}}}_{\text{ray}}\in \{\left|+\right\rangle \left|1\right\rangle ,\left|-\right\rangle \left|1\right\rangle ,\left|+\right\rangle \left|2\right\rangle ,\left|-\right\rangle \left|2\right\rangle \}$, a four-dimensional Hilbert space, while in the wave picture the emitted output from the laser is a single paraxial spatial mode. By using an astigmatic mode converter consisting of two cylindrical lenses^73^, the planar trajectory mode can be transformed into a skewed trajectory carrying OAM, as shown in Fig. 2c, d for the conversion technique and the resulting ray-wave vortex beam, respectively. Alongside the simulation are the measured beam profiles at selected propagation distances outside the cavity, as shown in Fig. 2e. To experimentally verify the ray-wave laser operation, we can use a lens to image the intra-cavity mode in free space and capture the transverse patterns at various propagation distances^72^. The result of this measurement for the $\left|{{\Omega }}=1/4\right\rangle$ state is shown in Fig. 3a. The results show the tell-tale signs of wave-like behaviour, as evident by the interference fringes with high visibility at *z* = 0 and *z* = *z*_R_, while appearing to be independent ray trajectories. Thus the laser output is a four-dimensional non-separable scalar state.

Since one of the DoFs is polarisation, our scheme requires us to engineer the cavity to achieve ray-dependent polarisation control without any intra-cavity elements. We achieve this by exploiting the fact that differing ray trajectories will impinge on the laser crystal at angles other than normal, as well as thermal effects as a function of the pump power (see Supplementary Information E). By deploying a c-cut crystal that exhibits angle-dependent birefringence at non-normal incident angles, we can mark our orbits with polarisation by simple adjustment of the pump light position and power, the results of which are shown in Fig. 3b. The visibility of the fringes decreases to zero as the pump power is increased (for a particular trajectory size) as a result of the orthogonal ray polarisation states. The power control of the gain results in all rays evolving from an entirely linear horizontal state to a vector state of diagonal ($\left|D\right\rangle$) and anti-diagonal ($\left|A\right\rangle$) polarisations. The results confirm that the cavity can be forced into a ray-like mode with different polarisations on lights of various ray orbits. By contrast, the prior vectorial ray-wave lasers allowed only the complete mode to be marked, where all rays were the same, limiting the state to 2D but multiple DoFs^62^. We derive a general analytical form for our output state (see Supplementary Information B); for the example geometry of Figs. 2 and 3, a laser mode in an eight-dimensional Hilbert space spanned by the basis states in ${{\mathcal{H}}}_{8}\in \left\{\left|+\right\rangle \left|1\right\rangle \left|D\right\rangle ,\left|-\right\rangle \left|1\right\rangle \left|D\right\rangle ,\left|+\right\rangle \left|2\right\rangle \left|D\right\rangle \right.$, $\left.\left|-\right\rangle \left|2\right\rangle \left|D\right\rangle ,\left|+\right\rangle \left|1\right\rangle \left|A\right\rangle ,\left|-\right\rangle \left|1\right\rangle \left|A\right\rangle ,\left|+\right\rangle \left|2\right\rangle \left|A\right\rangle ,\left|-\right\rangle \left|2\right\rangle \left|A\right\rangle \right\}$. We show the full states experimentally in Fig. 3c.

### Controlled generation of GHZ states

**Fig. 4:**
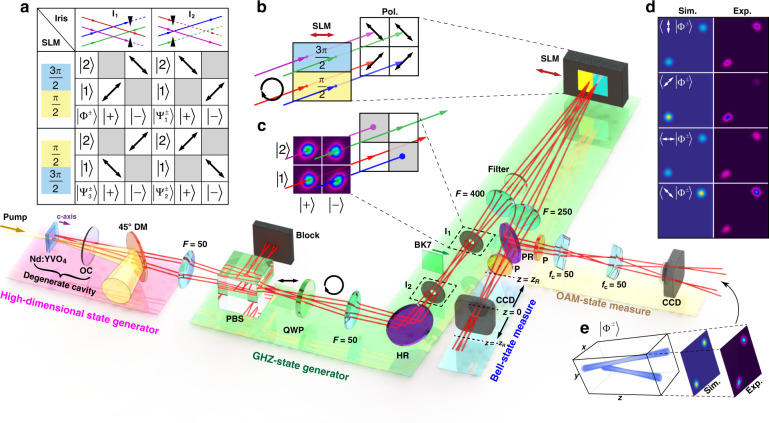
Creation of classical GHZ states. The experimental setup used to generate the classical GHZ states from our laser, including the core steps of high-dimensional state generation (laser), GHZ-state generation and two measurement steps to confirm the state properties. **a** Shows the required path and polarisation transformations needed for each GHZ state, performed by the iris (located at *I*_1_ or *I*_2_) and SLM phase masks (3*π*/2 and *π*/2), respectively. In **b** and **c**, this is unpacked graphically for the SLM modulation, altered in the polarisation of each ray state, and the iris modulation, where the incoming four lobes are reduced to two. **d** Shows the results for the vector beam corresponding to the first maximally entangled group $\left|{{{\Phi }}}^{\pm }\right\rangle$, obtained both experimentally (Exp.) and simulated (Sim.). The arrows depict the orientation of the polarizer in the measurement stage of the OAM-state measure. In the tomography measurement (Bell-state measure), each of the eight GHZ states can be inferred by just a polarizer and a CCD camera. The CCD camera is moved to different locations and captures the interferometric fringes for a visibility calculation. **e** The final trajectory in space of one of the GHZ states, showing the two-lobed structure. (OC output coupler mirror, DM dichroic mirror, PBS polarisation splitting prism, QWP quarter-wave plate, HR high-reflective mirror, PR partial-reflective mirror, SLM spatial light modulator, CCD charge-coupled device camera, P polarizer)

Next, we show that it is possible to controllably modulate this ray-wave vector beam to the desired states in a high-dimensional space. By way of example, we create the classical version of the famous GHZ states^74^, with the concept and setup shown in Fig. 4. In particular, we wish to create a complete set of tri-partite GHZ states, namely, $\left|{{{\Phi }}}^{\pm }\right\rangle$, $\left|{{{\Psi }}}_{1}^{\pm }\right\rangle$, $\left|{{{\Psi }}}_{2}^{\pm }\right\rangle$ and $\left|{{{\Psi }}}_{3}^{\pm }\right\rangle$, which construct the complete bases in eight-dimensional space, with full details given in Supplementary Information C.

We externally modulate the output state from the laser to engineer the amplitude and phase of each term independently, producing the general state

1 $$\begin{array}{lll}\left|{u}_{8}\right\rangle &=&{\alpha }_{1}\left|+\right\rangle \left|1\right\rangle \left|D\right\rangle +{\alpha }_{2}\left|-\right\rangle \left|1\right\rangle \left|D\right\rangle +{\alpha }_{3}\left|+\right\rangle \left|2\right\rangle \left|D\right\rangle \\ &&+\,{\alpha }_{4}\left|+\right\rangle \left|1\right\rangle \left|A\right\rangle +{\alpha }_{5}\left|-\right\rangle \left|2\right\rangle \left|D\right\rangle +{\alpha }_{6}\left|-\right\rangle \left|1\right\rangle \left|A\right\rangle \\ &&+\,{\alpha }_{7}\left|+\right\rangle \left|2\right\rangle \left|A\right\rangle +{\alpha }_{8}\left|-\right\rangle \left|2\right\rangle \left|A\right\rangle \end{array}$$

and converting it to each of the eight GHZ basis states. For example, the transformation

2 $$\left|{u}_{8}\right\rangle \to \left|{{{\Phi }}}^{\pm }\right\rangle =\frac{\left|+\right\rangle \left|1\right\rangle \left|D\right\rangle \pm \left|-\right\rangle \left|2\right\rangle \left|A\right\rangle }{\sqrt{2}}$$

requires a modulation that sets all amplitudes to zero except $| {\alpha }_{1}| =| {\alpha }_{8}| =\frac{1}{\sqrt{2}}$ and a relative phase shift between the two decomposed ray modes. The general setup to achieve this (and other modulations) is shown in Fig. 4. The main experimental arrangement includes the laser for creating the initial high-dimensional state, followed by a tailoring step to convert it into specific desirable classes, here the GHZ states are generated as an example. Finally, the states are directed to two measurement devices: the vectorial nature of the prepared states is measured by a polarizer and camera (OAM-state measure), and a Bell-state measurement device, which we introduce here, is used for the tomographic projections (see Methods section). Figure 4a–c graphically illustrate the transformations required. Using the $\left|{{{\Phi }}}^{\pm }\right\rangle$ state as an example (see full details for all cases in Methods section and Supplementary Information D), we switch from a linear polarisation basis to a circular one with a quarter-wave plate (QWP), eliminate light on the ray states $\left|+\right\rangle \left|2\right\rangle$ and $\left|-\right\rangle \left|1\right\rangle$ by iris *I*_1_, and then modulate the polarisation of the $\left|+\right\rangle \left|1\right\rangle$ and $\left|-\right\rangle \left|2\right\rangle$ paths into diagonal and anti-diagonal polarized states using programmed phases on a spatial light modulator (SLM). Similar transformations allow us to generate all GHZ states in the complete family of maximally entangled states. The controllable generation of all the GHZ states provides not only the ability to shape an on-demand high-dimensional structured beam but also the verification that the general SU(2) geometric beam in our system is indeed expressed in an eight-dimensional space, as the GHZ states form a complete basis in eight dimensions.

Notably, both the OAM state and planar state of the ray-wave structured beam play important roles in identifying high-dimensionality. For the OAM state, the mode carrying twisted ray-wave structures have a more effective ray-like effect, rather than the planar state, where the spatial twisted ray bundle does not interfere upon propagation, which enables us to clearly identify the polarisation on each individual ray. On the other hand, the planar ray trajectory has a wave interference region for the included rays, and the interferometric fringes allow us to identify the phase differences among the various rays. This is the basic principle to realise higher-dimensional control of the ray-wave structured beam rather than a common beam without ray-wave duality.

Experimental images of the twisted rays, together with the theoretical predictions, are shown in Fig. 4d for the $\left|{{{\Phi }}}^{\pm }\right\rangle$ states as a function of the orientation of the polarizer in the OAM-state measurement step, showing excellent agreement. The intensity distribution is a two-lobed structure, consistent with the corresponding GHZ state, while the evolution of the lobe intensities confirms the vectorial nature of the field. A reconstruction of the GHZ-state mode propagation in free-space is shown in Fig. 4e. The results for all other states are shown in Fig. S2 of the Supplementary Information.

**Fig. 5:**
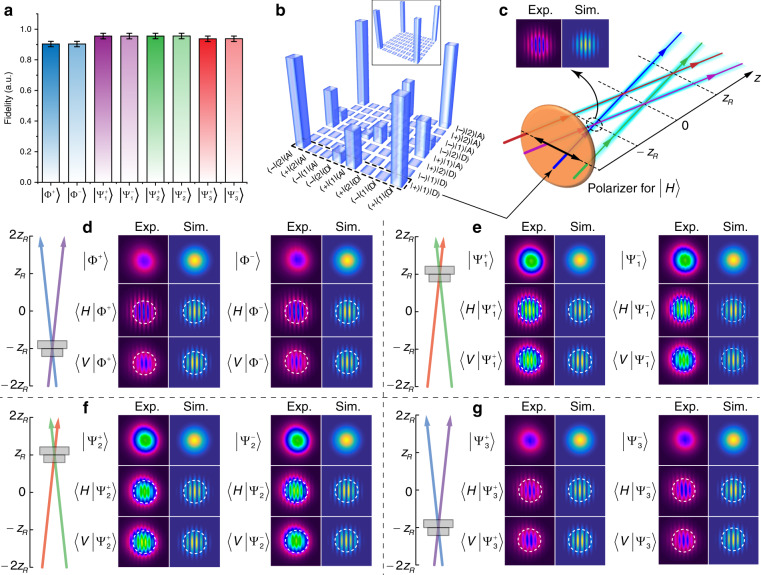
GHZ-state tomography. **a** Fidelity of each GHZ state, with error bars showing the standard error. **b** The experimental density matrix of the first GHZ state, $\left|{{{\Phi }}}^{+}\right\rangle$, in good agreement with the theoretical matrix (inset). This agreement is achieved by a new tomography based on visibility at specific *z* planes for each polarisation projection, illustrated in **c** for one projection, showing the interference pattern at the overlapping region of the two orbits corresponding to the GHZ state. Experimental (Exp.) and theoretical (Sim.) results are shown for the states of **d** $\left|{{{\Phi }}}^{\pm }\right\rangle$, **e** $\left|{{{\Psi }}}_{1}^{\pm }\right\rangle$, **f** $\left|{{{\Psi }}}_{2}^{\pm }\right\rangle$ and **g** $\left|{{{\Psi }}}_{3}^{\pm }\right\rangle$, and the illustration on the left for each panel shows the location of the CCD camera when capturing the interference fringes

To quantitatively infer the fidelity of our classical GHZ states, we introduce a new tomography based on Bell-state projections, with the fidelity results for all eight states shown in Fig. 5a. Each was calculated from a density matrix of the state, with the results of this for the $\left|{{{\Phi }}}^{+}\right\rangle$ state (as the lowest fidelity example) shown in Fig. 5b. All other density matrix results can be found in Fig. S3 of the Supplementary Information. We find that the theoretical (inset) and measured density matrices are in very good agreement, with fidelities of >90%. We measure these values by a new tomography of classical light fields: the two lobes of the GHZ-state beam overlap at the non-OAM planar state, projected onto the polarisation states and the visibility in the fringes is measured: a Bell-state projection (see details in Methods section and Supplementary Information D), as illustrated in Fig. 5c, with the details for each projection shown in Fig. 5d–g. This allows the state amplitudes and relative phases to be determined, and hence the GHZ states can be clearly distinguished. From the visibility of each projection in the tomographic measurement, the density matrix is inferred and the fidelity of each GHZ state is quantitatively calculated. For example, $\left|{{{\Phi }}}^{+}\right\rangle$ shows no fringes for the original state, a centre-bright fringe after projecting onto the $\left|H\right\rangle$ state i.e. for $\left\langle H| {{{\Phi }}}^{+}\right\rangle$, and a centre-dark fringe for $\left\langle V| {{{\Phi }}}^{+}\right\rangle$, similarly for all other examples of Fig. 5d–g.

## Discussion

Here, we have demonstrated vectorial structured light that is non-separable (classically entangled) in three DoFs and eight dimensions, beyond the existing laser limitation of two dimensions in polarisation structured vector beams. In addition to the arguably simple approaches for vectorially structured beams at the source, we design an external SLM modulation system to realise the complete control of on-demand states in higher-dimensional space, using the GHZ states as an example. The powerful classical GHZ states were proposed for quantum simulation in a classical optical system^57,58^, and some intriguing applications to quantum-like information protocols were realised^59–61^. However, the prior classical GHZ states were realised by complicated multi-beam optical system. It is still a challenge to control a full set of GHZ states in a paraxial structured beam. Here we reached this target and realised the first complete set of GHZ states in vectorial light beams. We introduced a new tomography approach for verifying high-dimensional states, which we used to quantify the fidelity of our generated GHZ states. In our experimental demonstration, we used a cavity to operate a four-ray SU(2) geometric vector beam in eight dimensions to simulate tri-partite entanglement.

Importantly, our method is easily scalable to achieve higher-dimensional (more than eight dimensions) control. In the above results, we used only ray-wave structured light in the degenerate state of $\left|{{\Omega }}=1/4\right\rangle$ to demonstrate our scheme. As such, the ray trajectory has an oscillation period of four round-trips inside the cavity with two oscillation direction states and two round-trip location states. For this reason, we can control this structured light in 2^3^ = 8-dimensional space. This choice is not peculiar, we can also choose cases of other frequency ratios such as $\left|{{\Omega }}=1/6\right\rangle$, the ray-wave mode of which has ray trajectories with a period of 6 in the cavity, thus, there are 3 location states, allowing control in a 2 × 3 × 2 = 12-dimensional space (see discussion on more general cases in Supplementary Information). Moreover, the ray-wave duality beam has the potential to realise even higher-dimensional and multi-partite states with extended DoFs. In addition to the three DoFs used above, we can also use the OAM to form non-separable states, realising four-partite entangled states. Additionally, due to the multi-singularity property of ray-wave structured light, the sub-OAM on the sub-ray region can also be used to extend to the five-partite state, see more examples and detailed demonstrations in Supplementary Information H.

These classical analogues to multi-partite high-dimensional quantum states have already been suggested for tasks such as quantum channel error estimation and correction^35^, super-resolution imaging^24^, metrology and sensing^27^, optical communication^30^ and quantum decoherence studies under easily controlled conditions^36^. Excitingly for fundamental physical studies, the interaction between spin and trajectory involved in the optical spin Hall effects^75^ and spin–orbit Hall effects^76^ can now be studied in new ways with these engineered spin marked trajectory-like modes. Finally, we also offer a complete theoretical framework for our vectorially structured light beams, providing a rich parameter space for extending control and dimensionality, and fostering new fundamental studies with structured light.

## Methods

### Creating GHZ states

The GHZ generation step is shown as part of Fig. 4. The relevant transformations for generating our GHZ states are executed in two consecutive steps, intensity modulation and dynamic phase (SLM) modulation. A graphical representation of the scheme is illustrated in Fig. 4a, showing the polarisation control (rows) and intensity modulation of the orbits (columns). The unpacked schematics for the SLM and intensity modulations are given in Fig. 4b, c. Each panel describes the required modulation to produce each of the desired GHZ states.

To achieve path (intensity) modulation, we use an iris strategically positioned at *I*_1_ or *I*_2_. We unpack this technique in Fig. 4c, showing an example for modulation at location *I*_1_. The four quadrants represent the full orbits, while the grey regions represent the filtered orbits. Due to the special spatial structure of the SU(2) geometric beam, the iris induces a path-dependent intensity modulation: when an iris with an appropriate aperture size is placed at the negative Rayleigh length position (*I*_1_ position), paths $\left|-\right\rangle \left|1\right\rangle$ and $\left|+\right\rangle \left|2\right\rangle$ can be blocked, resulting in a diagonal intensity pattern in the corresponding vortex SU(2) beam (see Fig. 4c). When applied at the positive Rayleigh length position (*I*_2_ position), $\left|+\right\rangle \left|1\right\rangle$ and $\left|-\right\rangle \left|2\right\rangle$ are blocked, resulting in an anti-diagonal intensity pattern of the vortex SU(2) geometric beam.

For polarisation modulation, we first convert all rays to circular polarisation with a QWP and direct them towards the SLM with a controlled location. This is illustrated graphically in Fig. 4b, where the four orbits are marked with (anti-)diagonal polarisation states, as shown by the arrows. The phase mask of the SLM is split into two parts, one to modulate the state $\left|1\right\rangle$ and the other to modulate the state $\left|2\right\rangle$. Since the beam is circularly polarised and the SLM is sensitive only to the horizontal component, the polarisation of the $\left|1\right\rangle$ and $\left|2\right\rangle$ states can be independently controlled by encoding phase retardations on each section of the SLM. When the encoded phase-step mask is set to *π*/2 and 3*π*/2 for the split screen, respectively, states $\left|1\right\rangle \left|D\right\rangle$ and $\left|2\right\rangle \left|A\right\rangle$ are produced. Likewise, when the mask is flipped (3*π*/2 and *π*/2), states $\left|1\right\rangle \left|A\right\rangle$ and $\left|2\right\rangle \left|D\right\rangle$ are produced. The combination of intensity and polarisation modulation results in four kinds of vector vortex beams corresponding to the four maximally entangled groups of three-partite GHZ states. Finally, the inter-modal phase between states is easily controlled with a thin BK7 plate as a phase retarder, which is partially inserted into the path of one ray group to add a phase difference of 0 or *π*.

### Bell-state projections

We describe the working principle of our Bell-state measurement approach, shown as part of Fig. 4, which comprises a focusing lens (*F* = 250 mm), polarizer and CCD camera. The camera can be placed at different regions within the focus of the lens.

Our measurement scheme exploits the properties of the multi-particle GHZ states. A GHZ state will be reduced to a two-particle Bell state after a superposition measurement of one of the particles (see Supplementary Information D). For the classical GHZ states, superposition projections of one of the DoFs leaves the remaining DoFs to collapse to maximally entangled Bell states (see Supplementary Information D for the full explanation). Here the polarisation DoF is chosen as a candidate to realise the Bell projection.

After projections onto the $\left|H\right\rangle$ and $\left|V\right\rangle$ states, the $\left|{{{\Phi }}}^{\pm }\right\rangle$ or $\left|{{{\Psi }}}_{3}^{\pm }\right\rangle$ states are reduced to the $\left|{\psi }^{\pm }\right\rangle$ and $\left|{\psi }^{\mp }\right\rangle$ states, $\left|{{{\Psi }}}_{1}^{\pm }\right\rangle$ or $\left|{{{\Psi }}}_{2}^{\pm }\right\rangle$ to $\left|{\phi }^{\pm }\right\rangle$ and $\left|{\phi }^{\mp }\right\rangle$ (refer to in Supplementary Information D and E). The “+” and “−” signals in the Bell states can be distinguished by the complementary interferometric fringes of the corresponding phase difference of 0 and *π* between two SU(2) ray-orbits. For measuring $\left|{{{\Phi }}}^{\pm }\right\rangle$ and $\left|{{{\Psi }}}_{3}^{\pm }\right\rangle$, the CCD camera can be located at the *z* = − *z*_R_ position, where $\left|-1\right\rangle$ and $\left|+2\right\rangle$ orbits overlap. For $\left|{{{\Psi }}}_{1}^{\pm }\right\rangle$ and $\left|{{{\Psi }}}_{2}^{\pm }\right\rangle$, the CCD camera should be located at the *z* = *z*_R_ position where the $\left|+1\right\rangle$ and $\left|-2\right\rangle$ orbits overlap. Without the polarisation projection, the pattern shows no fringes since the light on the corresponding two orbits is incoherent. After projection onto the $\left|H\right\rangle$ or $\left|V\right\rangle$ states, different interference fringes are observed for different reduced Bell states.

For the group $\left|{{{\Phi }}}^{\pm }\right\rangle$, the “±” cannot be distinguished by the intensity patterns. If we project the polarisation onto the $\left|H\right\rangle$ state to observe the pattern of $\left\langle H| {{{\Phi }}}^{\pm }\right\rangle$, the pattern of the original state $\left|{{{\Phi }}}^{\pm }\right\rangle$ will be reduced to the Bell states $\left|{\psi }^{\pm }\right\rangle$ and two different patterns of complementary fringes will be observed, centre-bright fringes for $\left|{\psi }^{+}\right\rangle$ (the original state should be $\left|{{{\Phi }}}^{+}\right\rangle$) and centre-dark fringes for $\left|{\psi }^{-}\right\rangle$ (the original state should be $\left|{{{\Phi }}}^{-}\right\rangle$). We can also use projected state $\left\langle V| {{{\Phi }}}^{\pm }\right\rangle$ to distinguish the “±”, such that $\left\langle V| {{{\Phi }}}^{+}\right\rangle$ should be centre-dark fringes corresponding to the Bell state $\left|{\psi }^{-}\right\rangle$ and $\left\langle V| {{{\Phi }}}^{-}\right\rangle$ should be centre-bright fringes corresponding to the Bell state $\left|{\psi }^{+}\right\rangle$. In the experiment, we can use a BK7 thin plate to cover one of the two orbits and rotate it slightly to control the phase difference between them to control a phase difference of *π*, switching from the “+” to the “−” state. Other GHZ states can be generated in a similar way, fulfilling a completed set in eight-dimensional Hilbert space.

### OAM measurement of the SU(2) geometric vortex beam

In contrast to the conventional vortex beam, the transverse pattern of which is always circularly symmetric (i.e., its pattern rotated around the central axis at an arbitrary angle coincides with itself), the SU(2) geometric vortex beam with SU(2) rotational symmetry (its pattern rotated around the central axis at a prescribed angle coincides with itself) can be directly observed in natural space, as the spatial twisted multi-ray trajectory manifests the OAM. The spatial twisted trajectories of an SU(2) geometric vortex beam can be directly measured by the scanning of a CCD camera, without other techniques such as interferometry of the conventional OAM measurement. Thus, the steps for the OAM measurement for an SU(2) geometric vortex beam are as follows: we first identify the beam trajectory by scanning the spatial distribution with a CCD device along the *z*-axis after the astigmatic lens converter, at a range from beam focus to Rayleigh length. Through this process, we obtain the 3D distribution of the beam which is characterised by a twisted trajectory with four-ray-paths. The four-lobe pattern located on the corners of a square shape angularly rotates along the *z*-axis with the increase in propagation distance. We can numerically compare the experimental twisted trajectory to a variety of OAM values and draw a correlation between the experimental and simulated results, as shown in Fig. S6 of the Supplementary Information. Additionally, the topological charge of the OAM can be quantitatively measured experimentally. To achieve this, we apply a triangular truncated aperture located at the vortex centre, and then observe the far-field diffraction pattern, that emerges as a pattern of optical lattices, and the value of topological charges in the measured SU(2) geometric vortex beam is directly related to the spot number in the diffracting optical lattices^77^. See the detailed process of this measurement in Supplementary Information G.

## Supplementary information

Supplementary Information: Creation and control of high-dimensional multi-partite classically entangled light
