# Supplementary material for: Creation and control of high-dimensional multi-partite classically entangled light
Source: Light Sci Appl. 2021 Mar 8;10:50. doi: 10.1038/s41377-021-00493-x (PMC7940607; doi:10.1038/s41377-021-00493-x)
Supplement: Supplementary file 1 — Supplementary Information: Creation and control of high-dimensional multi-partite classically entangled light [file 41377_2021_493_MOESM1_ESM.pdf]

Supplementary Information: Creation and control of high-dimensional multi-partite classically entangled light

Yijie Shen[1,2,3], Isaac Nape[1], Xilin Yang[4], Xing Fu[2,5], Mali Gong[2,5], Darryl Naidoo[1,6], and Andrew Forbes[1]

[1] *School of Physics, University of the Witwatersrand, Private Bag 3, Wits 2050, South Africa*

[2] *State Key Laboratory of Precision Measurement Technology and Instruments, Department of Precision Instrument, Tsinghua University, Beijing 100084, China*

[3] *(Present address) Optoelectronics Research Centre, University of Southampton, Southampton SO17 1BJ, UK*

[4] *Electrical and Computer Engineering Department, University of California, Los Angeles, CA 90095, USA*

[5] *Key Laboratory of Photonic Control Technology (Tsinghua University), Ministry of Education, Beijing 100084, China*

[6] *CSIR National Laser Centre, PO Box 395, Pretoria 0001, South Africa*

(Dated: January 8, 2021)

A. State representation of ray-wave duality mode

In this section, we introduce fundamental notions of ray-wave duality, SU(2) coherent states, and geometric modes for the benefit of the reader, Refs [1–5] are related for further reading about the theoretical background.

It is known that when a laser cavity is operated in a special geometry, the mode appears to be the result of a ray-like periodic orbit forming a closed path that is ever repeating, namely the ray-wave duality [1–5]. Here we give a representation of such mode by the oscillating direction states and round-trip location states.

Based on geometrical optics, the ABCD matrix is used to characterize the propagation of optical ray trajectories inside a stable plano-concave cavity. Since it was proved that the cavity length satisfies $L/R = \sin^2(\Omega\pi)$ [3, 6], where $\Omega = P/Q = \Delta f_T/\Delta f_L$ (Δf_T and Δf_L are transverse and longitudinal mode frequency spacings, integers P and Q are coprime), the corresponding ABCD matrix is given by [6]:

$$\mathbf{A} = \begin{bmatrix} 1-\frac{2L}{R} & 2L\left(1-\frac{L}{R}\right) \\ -\frac{2}{R} & 1-\frac{2L}{R} \end{bmatrix} = \begin{bmatrix} \cos(2\Omega\pi) & \frac{R}{2}\sin^2(2\Omega\pi) \\ -\frac{2}{R} & \cos(2\Omega\pi) \end{bmatrix}. \tag{S.1}$$

After n times of round trips in the frequency-degenerate cavity, the matrix is derived as:

$$\mathbf{A}^n = \begin{bmatrix} \cos(2n\Omega\pi) & \frac{R}{2}\sin^2(2n\Omega\pi) \\ -\frac{2}{R}\frac{\sin(2n\Omega\pi)}{\sin(2\Omega\pi)} & \cos(2n\Omega\pi) \end{bmatrix}. \tag{S.2}$$

Because $Q\Omega = P$ is an integer, $\cos(2Q\Omega\pi) = 1$, $\sin(2Q\Omega\pi) = 0$, and Q-th power of $\mathbf{A}$ is an unit matrix:

$$\mathbf{A}^Q = \begin{bmatrix} \cos(2Q\Omega\pi) & \frac{R}{2}\sin^2(2Q\Omega\pi) \\ -\frac{2}{R}\frac{\sin(2Q\Omega\pi)}{\sin(2\Omega\pi)} & \cos(2Q\Omega\pi) \end{bmatrix} = \mathbf{I}. \tag{S.3}$$

Equation (S.3) reveals that an optical ray oscillating at an arbitrary position within the cavity would coincide exactly with the initial state after Q times of round trips. Therefore, it is proved that the lasing modes have a preference to be localized on the periodic ray trajectories in a frequency-degenerate state $|\Omega = P/Q\rangle$, a special case of $|\Omega = 1/4\rangle$ are shown in Fig. S1. Manifested by the ray matrix, the parametric equation for each periodic orbit in SU(2) oscillation can be derived. For the planar geometric modes, the orbits can be derived as [2]:

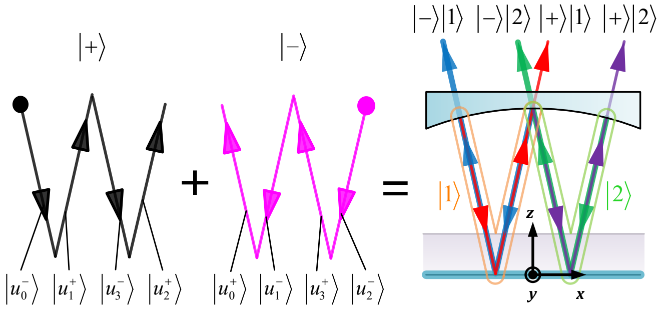

FIG. S1. **Trajectory direction and location states.** The ray orbit components for the positive and negative x-direction construct the trajectory for the direction states $|+\rangle$ and $|-\rangle$, and the two $|+\rangle$ and $|-\rangle$ construct a complete oscillation in a degenerate laser cavity. The output ray states can be represented by the product states of direction and location states.

$$\begin{aligned} x_s^{\pm}(z) &= \sqrt{N}w_0\left[\cos(\theta_s+\phi_x) \mp (z/z_R)\sin(\theta_s+\phi_x)\right] \\ &= \sqrt{N}w(z)\cos\left[\theta_s+\phi_x \pm \vartheta(z)\right] \end{aligned} \tag{S.4}$$

where $\theta_s = (P/Q)2\pi s$, $s = 0, 1, 2, \cdots, Q-1$ is the running index for the different rays, ϕ_x is the phase factor related to the initial position and direction, $\sqrt{N}$ determines the transverse size of the ray trajectory, and $+$ and $-$ in the symbol of $\pm$ indicate the backward and forward rays, respectively. Defining the dimensionless variable $\widetilde{x} = \sqrt{2}x/w(z)$, the expression for the ray equation can be expressed as $\widetilde{x}(z) = \mathrm{Re}[\sqrt{2}u_s^{\pm}(z)]$ with:

$$u_s^{\pm}(z) = \sqrt{N}\mathrm{e}^{-\mathrm{i}[\theta_s+\phi_x\pm\vartheta(z)]}. \tag{S.5}$$

when $\phi_x = n\pi/Q$ ($n \in \mathbb{Z}$), the forward and backward rays would be coincidently overlapped (the forward and backward is at longitudinal z-direction), and the bouncing orbits with positive and negative transverse directions share the same location.

For example at $|\Omega = 1/4\rangle$ ($\phi_x = \pi/4$) we used in the main text, positive and negative (to x-axis) W-shaped

trajectories coincide exactly, i.e. the positive and negative oscillating states are expressed by:

$$|+\rangle = |u_0^-\rangle|u_1^+\rangle|u_3^-\rangle|u_2^+\rangle, \quad |-\rangle = |u_0^+\rangle|u_1^-\rangle|u_3^+\rangle|u_2^-\rangle, \tag{S.6}$$

as shown in Fig. 2 in the main text. Additionally, in the four periodic round-trips, two share the first bouncing location and other two share the second one, i.e. the round-trip location states are expressed by:

$$|1\rangle = |u_0^\pm\rangle|u_1^\pm\rangle, \quad |2\rangle = |u_2^\pm\rangle|u_3^\pm\rangle. \tag{S.7}$$

Each ray state is related to the round trip locations and oscillation directions, which can be given by:

$$\begin{aligned} |+\rangle|1\rangle &= |u_1^+\rangle, \quad |+\rangle|2\rangle = |u_2^+\rangle, \\ |-\rangle|1\rangle &= |u_0^+\rangle, \quad |-\rangle|2\rangle = |u_3^+\rangle. \end{aligned} \tag{S.8}$$

The ray trajectories in a complete oscillation and the output ray states from cavity are shown in Fig. S1. In our setup, the concave mirror is the laser output mirror, thus only the forward-propagation states are observable, as the four output ray states of $|-\rangle|1\rangle$, $|-\rangle|2\rangle$, $|+\rangle|1\rangle$, $|+\rangle|2\rangle$, marked by blue, green, red, and purple, in Fig. S1.

For the general spatial geometric mode with OAM, the equations for the 3D skewed rays can be written as [2]:

$$\begin{cases} u_s^\pm(z) = \sqrt{N_x}\mathrm{e}^{-\mathrm{i}[\theta_s+\phi_x\pm\vartheta(z)]} \\ v_s^\pm(z) = \sqrt{N_y}\mathrm{e}^{-i[\theta_s+\phi_y\pm\vartheta(z)]} \end{cases} \tag{S.9}$$

where $\theta_s = (P/Q)2\pi s$, $s = 0, 1, 2, \cdots, Q-1$, and the dimensionless variable in the y-direction is similarly defined as $\widetilde{y} = \sqrt{2}y/w(z)$ with the ray equation $\widetilde{y}(z) = \mathrm{Re}[\sqrt{2}v_s^\pm(z)]$, here $+$ and $-$ in the symbol of $\pm$ indicate the positive and negative OAM states (the spatial trajectory is right-hand or left-hand twisted), they together constitute a completed oscillation in cavity. For constituting a completed oscillation with both OAM states in a cavity, the phase factors yield $|\phi_x - \phi_y| = \pi/2$.

The above is the ray representation of geometric modes. Then, we need to derive the wave representation coupled with the geometric ray trajectory. The Gaussian wave packet with the central peak moving along the path $x = \mathrm{Re}(\sqrt{2}u) = \sqrt{2N}\cos(\omega t + \varphi_0)$ can be derived as [2]:

$$\pi^{-1/4}\mathrm{e}^{-x^2/2}F(x,u) = \sum_{n=0}^{\infty} a_n\psi_n(x)\mathrm{e}^{-\mathrm{i}n(\omega t+\varphi_0)}, \tag{S.10}$$

where coefficients $a_n = N^{n/2}e^{-N/2}/\sqrt{n!} = \sqrt{P(n,N)}$, where $P(n,N)$ is the Poisson distribution, $F(x,u) = \mathrm{e}^{-\left(u^2+|u|^2-2\sqrt{2}ux\right)/2}$ is the wave function coupled to trajectory of u, and $\psi_n(x)$ is the nth-order Hermite–Garussian (HG) function. The wave representation of a Gaussian wave packet moving along the s-th ray in a spatial geometric mode can be given by [2]:

$$\Phi(\mathbf{r}, u_s^\pm, v_s^\pm) = G(\mathbf{r})F(\widetilde{x}, u_s^\pm)F(\widetilde{y}, v_s^\pm). \tag{S.11}$$

where $G(\mathbf{r}) = \pi^{-1/2}\mathrm{e}^{-(\widetilde{x}^2+\widetilde{y}^2)(1+\mathrm{i}\widetilde{z})/2}\mathrm{e}^{\mp\mathrm{i}\vartheta(\widetilde{z})}$ represents the fundamental mode Gaussian beam and $\mathbf{r} = (x, y, z)$ the Cartesian coordinates. In terms of Eq. (S.11), the resonant mode for the forward and backward components of a complete period is given by [2]:

$$\Psi_{N_x,N_y}^\pm(\mathbf{r}) = \sum_{s=0}^{Q-1} \Phi\left(\mathbf{r}, u_s^\pm, v_s^\pm\right)\mathrm{e}^{\mathrm{i}(N_x+N_y)\theta_s}, \tag{S.12}$$

where the phase term $\mathrm{e}^{\mathrm{i}(N_x+N_y)\theta_s}$ is associated with the transverse frequency. For $N_x = 0$ or $N_y = 0$, Eq. (S.12) represents the planar geometric modes with ray structure on (x, z) or (y, z) plane; for $N_x = N_y \neq 0$, circular vortex geometric modes; for $0 \neq N_x \neq N_y \neq 0$, elliptical vortex geometric modes. In the above description, N_x or N_y should be large enough to stimulate the ray-like properties, otherwise the wave-like property is superior and the pattern will be nearly a certain eigenmode.

Hereinafter, we demonstrate the wave representation fulfills the wave-packet form of SU(2) coherent state. Using planar trajectory $N_x = N$ and $N_y = 0$ for convenience, it can be obtained that $v_s^\pm = 0$ and $F(\widetilde{y}, 0) = 1$, and the planar geometric mode is given by [2]:

$$\Psi_N^\pm(\mathbf{r}) = \sum_{s=0}^{Q-1} G(\mathbf{r})F\left(\widetilde{x}, u_s^\pm\right)\mathrm{e}^{\mathrm{i}N\theta_s}, \tag{S.13}$$

Substituting Eq. (S.10) into Eq. (S.13) and ignoring the constant coefficient, we get:

$$\begin{aligned} \Psi_N^\pm(\mathbf{r}) &\propto \sum_{s=0}^{Q-1} G(\mathbf{r})\mathrm{e}^{\frac{\widetilde{x}^2}{2}} \sum_{n=0}^{\infty} a_n\psi_n(\widetilde{x})\mathrm{e}^{-\mathrm{i}n[\theta_s+\phi_x\pm\vartheta(z)]}\mathrm{e}^{\mathrm{i}N\theta_s} \\ &= \sum_{s=0}^{Q-1}\sum_{n=0}^{\infty} a_n\psi_n(\widetilde{x})\mathrm{e}^{\frac{\widetilde{x}^2}{2}}G(\mathbf{r})\mathrm{e}^{\mp\mathrm{i}n\vartheta(z)-\mathrm{i}n(\theta_s+\phi_x)+\mathrm{i}N\theta_s} \\ &\propto \sum_{s=0}^{Q-1}\sum_{n=0}^{\infty} a_n\psi_{n,0,l_n}^{(\mathrm{HG})}(x,y,\pm z)\mathrm{e}^{-\mathrm{i}n\phi_x}\mathrm{e}^{\mathrm{i}(N-n)\theta_s} \\ &= \sum_{n=0}^{\infty} a_n\psi_{n,0,l_n}^{(\mathrm{HG})}(x,y,\pm z)\mathrm{e}^{-\mathrm{i}n\phi_x}\sum_{s=0}^{Q-1}\mathrm{e}^{\mathrm{i}(N-n)\theta_s}, \end{aligned} \tag{S.14}$$

where $\psi_{n,m,l}^{(\mathrm{HG})}(x,y,z)$ represents the HG mode with transverse mode indices of (n,m) and longitudinal mode index of l. The indices of the decomposed HG modes in Eq. (S.14) should fulfills the frequency-degenerate condition, $n = n_0 + KQ$ and $l_n = l_0 - KP$ [3]. Setting $n' = N - n$, the last term in the external summation notation of Eq. (S.14) can be written as:

$$\sum_{s=0}^{Q-1}\mathrm{e}^{\mathrm{i}n'\theta_s} = \sum_{s=0}^{Q-1}\mathrm{e}^{\mathrm{i}n'\frac{P}{Q}2\pi s}. \tag{S.15}$$

When $n' = KQ$ ($K \in \mathbb{Z}$), $\mathrm{e}^{\mathrm{i}n'\frac{P}{Q}2\pi s} = \mathrm{e}^{\mathrm{i}2\pi KPs} = 1$ and Eq. (S.15) is equal to a constant Q; when $n' \neq KQ$,

Eq. (S.15) is always a sum of the complex numbers uniformly distributed on the unit circle of the complex plane, thus it should be zero. And then, we use the Dirac notation to represent the spatial mode, and note the set of frequency-degenerate HG modes with number of M as $|K,M\rangle = \left|\psi^{(\mathrm{HG})}_{n_0+KQ,0,l_0-KP}\right\rangle$, Eq. (S.14) can be simplified and given as:

$$\begin{aligned}|\Psi^{\pm}_{N}(\mathbf{r})\rangle &\propto \sum_{K=0}^{M} a_{n_0+KQ}\mathrm{e}^{-\mathrm{i}KQ\phi_x}\left|\psi^{(\mathrm{HG})}_{n_0+KQ,0,l_0-KP}\right\rangle \\ &= \sum_{K=0}^{M}\sqrt{P(n_0+KQ,M)}\mathrm{e}^{-\mathrm{i}KQ\phi_x}|K,M\rangle \\ &\propto \sum_{K=0}^{M}\sqrt{B(K;M,\frac{1}{2})}\mathrm{e}^{-\mathrm{i}KQ\phi_x}|K,M\rangle \\ &= \frac{1}{2^{M/2}}\sum_{K=0}^{M}\binom{M}{K}^{1/2}\mathrm{e}^{\mathrm{i}K\phi}\langle x,y|K,M\rangle, \quad \text{(S.16)}\end{aligned}$$

where the coherent state phase $\phi = Q\phi_x$, and the Poisson distribution $P(n_0+KQ,M)$ is approximated by Binomial distribution $B(K;M,1/2)$ when $M = 4Q^2n_0$ is large enough, where $B(k;n,p) = \binom{n}{k}p^k(1-p)^{n-k}$ is Binomial distribution, according to the central-limit theorem. Then the laser mode Eq. (S.16) shares the same form of SU(2) coherent state as [7]:

$$|\phi\rangle = \frac{1}{2^{M/2}}\sum_{K=0}^{M}\binom{M}{K}^{1/2}\mathrm{e}^{\mathrm{i}K\phi}\,|K,M\rangle. \quad \text{(S.17)}$$

Therefore, the laser wave-packet in frequency-degenerate cavity has the formation of SU(2) coherent state, namely SU(2) geometric mode, with the property of ray-wave duality, the laser mode can be not only characterized by the wave function representation but also coupled with classical oscillating trajectory given by ray representation. Similarly, if we chose the eigenstates of $|K,M\rangle$ as a set of frequency-degenerate Laguerre-Gaussian (LG) mode, the SU(2) coherent state represents the ray-wave geometric vortex beam [8, 9].

B. General SU(2) geometric vector beams

Here we proposed a new generalized expression of SU(2) geometric vector beams. A normal SU(2) geometric beam can be modulated into a vector beam where arbitrary amplitude, phase, and polarisation for each ray orbit are tailored. By modifying Eq. (S.12), such a general vector beam can be given by:

$$\mathbf{\Psi}^{\pm}_{N_x,N_y}(\mathbf{r}) = \sum_{s=0}^{Q-1} A_s\mathrm{e}^{\mathrm{i}\phi_s}\mathbf{J}_s\Phi^{(s)}_{N_x,N_y}(\mathbf{r}), \quad \text{(S.18)}$$

where we set $\Phi^{(s)}_{N_x,N_y} = \Phi\left(\mathbf{r},u_s^{\pm},v_s^{\pm}\right)\mathrm{e}^{\mathrm{i}(N_x+N_y)\theta_s}$ for convenience; A_s, ϕ_s, and $\mathbf{J}_s$ are the amplitude, phase, and polarisation Jones vector of light at the s-th orbit.

When an SU(2) vector beam was controlled into ray-like state with large enough N_x and N_y without interference among lights on sub-orbits, the wavefunction along different orbits are independent without interference to each other, thus intensity pattern can be simplified as:

$$\left\|\mathbf{\Psi}^{\pm}_{N_x,N_y}(\mathbf{r})\right\|^2 = \sum_{s=0}^{Q-1} A_s^2\left|\Phi^{(s)}_{N_x,N_y}(\mathbf{r})\right|^2, \quad \text{(S.19)}$$

where $\|\mathbf{\Psi}\|$ is the Frobenius norm of vector $\mathbf{\Psi}$. Hereinafter, we derive the expression for the polarisation projection states. The Jones matrix for a linear polarizer with a inclined angle of θ_P is $\mathbf{J}_P = \begin{bmatrix}\cos\theta_P & 0\\ 0 & \sin\theta_P\end{bmatrix}$, which can project the light into the linear polarisation state with inclined angle of θ_P. The SU(2) geometric vector beam after projection can be given by:

$$\widetilde{\mathbf{\Psi}}^{\pm}_{N_x,N_y}(\mathbf{r}) = \sum_{s=0}^{Q-1} A_s\mathrm{e}^{\mathrm{i}\phi_s}\mathbf{J}_P\mathbf{J}_s\Phi^{(s)}_{N_x,N_y}(\mathbf{r}). \quad \text{(S.20)}$$

When N_x and N_y are both large enough, the lights on various orbits cannot make interference to each other, the intensity pattern can be given by:

$$\left\|\widetilde{\mathbf{\Psi}}^{\pm}_{N_x,N_y}(\mathbf{r})\right\|^2 = \sum_{s=0}^{Q-1} A_s^2\left\|\mathbf{J}_P\mathbf{J}_s\right\|^2\left|\Phi^{(s)}_{N_x,N_y}(\mathbf{r})\right|^2. \quad \text{(S.21)}$$

The SU(2) geometric vector beam can be expressed in the high-dimensional Hilbert space using the new DoFs from ray-wave representation. For instance of the geometric mode in degenerate state $|\Omega = 1/4\rangle$, the geometric vector beam can be represented by trajectory states:

$$\begin{aligned}\left|\mathbf{\Psi}^{\pm}_{N_x,N_y}\right\rangle &= \sum_{s=0}^{3} A_s\mathrm{e}^{\mathrm{i}\phi_s}\left|\mathbf{J}_s\right\rangle\left|\Phi^{(s)}_{N_x,N_y}\right\rangle \\ &= A_0\mathrm{e}^{\mathrm{i}\phi_0}\left|\mathbf{J}_0\right\rangle|-\rangle|1\rangle + A_1\mathrm{e}^{\mathrm{i}\phi_1}\left|\mathbf{J}_1\right\rangle|+\rangle|1\rangle \\ &\quad + A_2\mathrm{e}^{\mathrm{i}\phi_2}\left|\mathbf{J}_2\right\rangle|+\rangle|2\rangle + A_3\mathrm{e}^{\mathrm{i}\phi_3}\left|\mathbf{J}_3\right\rangle|-\rangle|2\rangle, \end{aligned} \quad \text{(S.22)}$$

where the state of polarisation $|\mathbf{J}_s\rangle$ is two dimensions, which can be linearly expanded by bases of $|D\rangle$ and $|A\rangle$. Therefore, the SU(2) vector beam of Eq. S.22 is expressed in 8-dimensional space with bases of $|+\rangle|1\rangle|D\rangle$, $|-\rangle|1\rangle|D\rangle$, $|+\rangle|2\rangle|D\rangle$, $|+\rangle|1\rangle|A\rangle$, $|-\rangle|2\rangle|D\rangle$, $|-\rangle|1\rangle|A\rangle$, $|+\rangle|2\rangle|A\rangle$, and $|+\rangle|2\rangle|A\rangle$.

We can control the SU(2) geometric beam into ray-like state and then make special modulations to realize various classical GHZ states [see Eqs. (S.32) to (S.35) in section C] as specific cases:

- $|\Phi^+\rangle$: $A_0 = A_2 = \frac{1}{\sqrt{2}}$, $A_1 = A_3 = 0$, $\phi_0 = \phi_2$, $|\mathbf{J}_0\rangle = |D\rangle$ and $|\mathbf{J}_2\rangle = |A\rangle$;

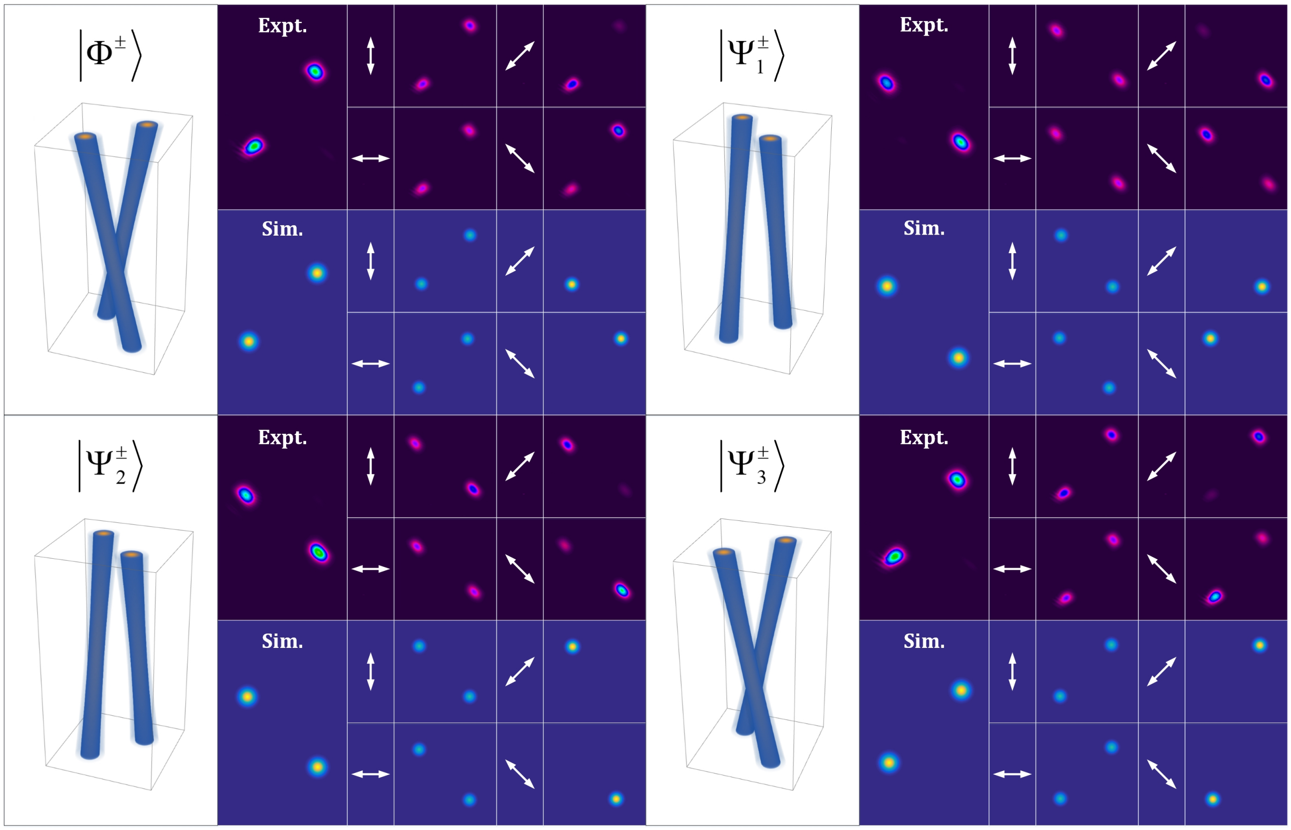

FIG. S2. **Control of four maximumly entangled groups in GHZ states.** The experimental and theoretical results of four general SU(2) vector beams corresponding to the four maximumly entangled groups in classical GHZ states. The white arrow means the allowed polarisation orientation of the polarizer. The plot on the left for each group shows the theoretical spatial wave-packet of the corresponding SU(2)-like vector beams. $|\Phi^{\pm}\rangle$: the SU(2)-like vector beam with diagonal intensity pattern where $|+1\rangle$ orbit with diagonal polarisation $|-2\rangle$ orbit with anti-diagonal polarisation; $|\Psi_1^{\pm}\rangle$: anti-diagonal intensity pattern, $|-1\rangle$ orbit with diagonal polarisation $|+2\rangle$ orbit with anti-diagonal polarisation; $|\Psi_2^{\pm}\rangle$: anti-diagonal intensity pattern, $|+2\rangle$ orbit with diagonal polarisation $|-1\rangle$ orbit with anti-diagonal polarisation; $|\Psi_3^{\pm}\rangle$: diagonal intensity pattern, $|-2\rangle$ orbit with diagonal polarisation $|+1\rangle$ orbit with anti-diagonal polarisation.

- $|\Phi^-\rangle$: $A_0 = A_2 = \frac{1}{\sqrt{2}}$, $A_1 = A_3 = 0$, $\phi_0 = \phi_2 + \pi$, $|\mathbf{J}_0\rangle = |D\rangle$ and $|\mathbf{J}_2\rangle = |A\rangle$;
- $|\Psi_1^+\rangle$: $A_1 = A_3 = \frac{1}{\sqrt{2}}$, $A_0 = A_2 = 0$, $\phi_1 = \phi_3$, $|\mathbf{J}_1\rangle = |D\rangle$ and $|\mathbf{J}_3\rangle = |A\rangle$;
- $|\Psi_1^-\rangle$: $A_1 = A_3 = \frac{1}{\sqrt{2}}$, $A_0 = A_2 = 0$, $\phi_1 = \phi_3 + \pi$, $|\mathbf{J}_1\rangle = |D\rangle$ and $|\mathbf{J}_3 = |A\rangle\rangle$;
- $|\Psi_2^+\rangle$: $A_0 = A_3 = \frac{1}{\sqrt{2}}$, $A_1 = A_2 = 0$, $\phi_0 = \phi_3$, $|\mathbf{J}_0\rangle = |D\rangle$ and $|\mathbf{J}_3\rangle = |A\rangle$;
- $|\Psi_2^-\rangle$: $A_0 = A_3 = \frac{1}{\sqrt{2}}$, $A_1 = A_2 = 0$, $\phi_0 = \phi_3 + \pi$, $|\mathbf{J}_0\rangle = |D\rangle$ and $|\mathbf{J}_3\rangle = |A\rangle$;
- $|\Psi_3^+\rangle$: $A_0 = A_2 = \frac{1}{\sqrt{2}}$, $A_1 = A_3 = 0$, $\phi_0 = \phi_2$, $|\mathbf{J}_0\rangle = |A\rangle$ and $|\mathbf{J}_2\rangle = |D\rangle$;
- $|\Psi_3^-\rangle$: $A_0 = A_2 = \frac{1}{\sqrt{2}}$, $A_1 = A_3 = 0$, $\phi_0 = \phi_2 + \pi$, $|\mathbf{J}_0\rangle = |A\rangle$ and $|\mathbf{J}_2\rangle = |D\rangle$;

The 3-D intensity patterns of various GHZ states with various polarisation projection states ($\theta_P = 0,\ \pi/2,\ \pi$, and $3\pi/2$) are shown in Fig. S2, where we set $N_x = N_y$ in simulation. When the vortex geometric mode is reduced into planar geometric mode, i.e. N_x or $N_y = 0$, we can directly use intensity pattern $\langle\Psi^{\pm}_{N_x,N_y}|\Psi^{\pm}_{N_x,N_y}\rangle$ to observe the interference effect among sub-orbits, which reveals the phase differences among the sub-orbits, as shown in Fig. 5 in the main article for various GHZ states. By experimentally measuring the amplitude, phase, and polarisation of each orbit (the phase can be reconstructed by the interference pattern among various basic states), we can evaluate the experimental state:

$$\begin{aligned}|\psi_{exp}\rangle = \alpha_1 |+\rangle |1\rangle |D\rangle + \alpha_2 |-\rangle |1\rangle |D\rangle + \alpha_3 |+\rangle |2\rangle |D\rangle \\ + \alpha_4 |+\rangle |1\rangle |A\rangle + \alpha_5 |-\rangle |2\rangle |D\rangle + \alpha_6 |-\rangle |1\rangle |A\rangle \\ + \alpha_7 |+\rangle |2\rangle |A\rangle + \alpha_8 |-\rangle |2\rangle |A\rangle , \end{aligned} \quad \text{(S.23)}$$

reconstitute the density matrix $\hat{\rho}_{exp} = |\psi_{exp}\rangle\langle\psi_{exp}|$ for each experimental GHZ state, and calculate the fidelities $F = \langle\Phi^{\pm}|\hat{\rho}_{exp}|\Phi^{\pm}\rangle$ and $F = \langle\Psi_i^{\pm}|\hat{\rho}_{exp}|\Psi_i^{\pm}\rangle$ $(i = 1, 2, 3)$ comparing with the theoretical density matrices of GHZ states. The theoretical and experimental tomography of density matrices for various GHZ states are shown in Fig. S3.

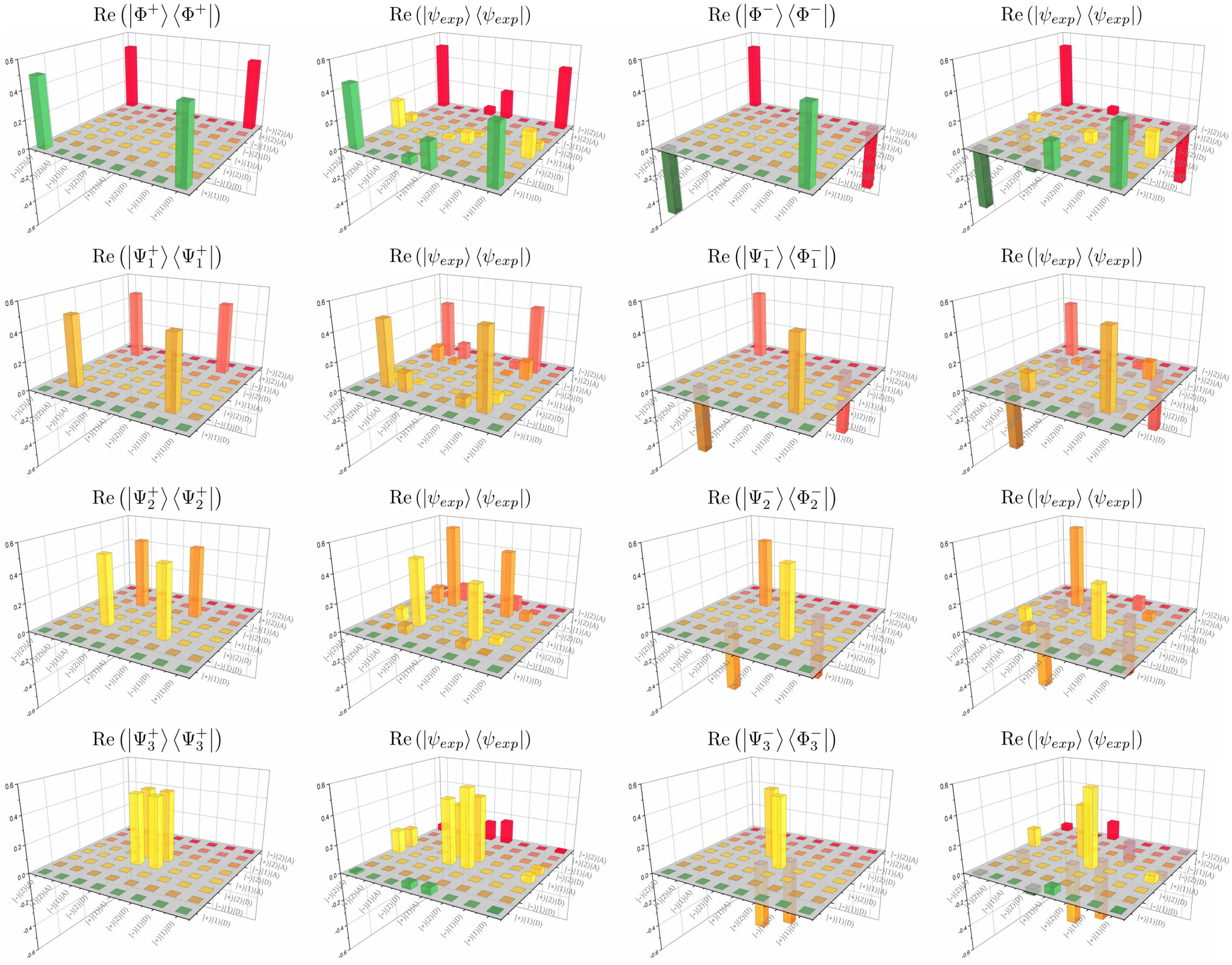

FIG. S3. **State tomography.** The theoretical and experimental results of the tomography of density matrices for various GHZ states.

C. Classical GHZ states

Maximally entangled states of two particles are called Bell states, acting as 4 eigenstates of 4-D Hilbert space, and those of N ($N \geq 3$) particles are called GHZ states, acting as 2^N eigenstates of 2^N-D Hilbert space [10]. The 4 Bell states in 2 maximumly entangled groups for polarisation-entangled photon pair are given by [11]:

$$\left|\phi^{\pm}\right\rangle = \frac{\left|H\right\rangle\left|V\right\rangle \pm \left|V\right\rangle\left|H\right\rangle}{\sqrt{2}}, \tag{S.24}$$

$$\left|\psi^{\pm}\right\rangle = \frac{\left|H\right\rangle\left|H\right\rangle \pm \left|V\right\rangle\left|V\right\rangle}{\sqrt{2}}. \tag{S.25}$$

They can linearly express a general 4-D polarisation-entangled state as bases:

$$\left|\psi\right\rangle = \alpha\left|H\right\rangle\left|H\right\rangle + \beta\left|H\right\rangle\left|V\right\rangle + \gamma\left|V\right\rangle\left|V\right\rangle + \delta\left|V\right\rangle\left|V\right\rangle. \tag{S.26}$$

The 8 GHZ states in 4 maximumly entangled groups for three-photon polarisation-entanglement are given by [10]:

$$\left|\Phi^{\pm}\right\rangle = \frac{\left|H\right\rangle\left|H\right\rangle\left|H\right\rangle \pm \left|V\right\rangle\left|V\right\rangle\left|V\right\rangle}{\sqrt{2}}, \tag{S.27}$$

$$\left|\Psi_1^{\pm}\right\rangle = \frac{\left|V\right\rangle\left|H\right\rangle\left|H\right\rangle \pm \left|H\right\rangle\left|V\right\rangle\left|V\right\rangle}{\sqrt{2}}, \tag{S.28}$$

$$\left|\Psi_2^{\pm}\right\rangle = \frac{\left|H\right\rangle\left|V\right\rangle\left|H\right\rangle \pm \left|V\right\rangle\left|H\right\rangle\left|V\right\rangle}{\sqrt{2}}, \tag{S.29}$$

$$\left|\Psi_3^{\pm}\right\rangle = \frac{\left|H\right\rangle\left|H\right\rangle\left|V\right\rangle \pm \left|V\right\rangle\left|V\right\rangle\left|H\right\rangle}{\sqrt{2}}. \tag{S.30}$$

They can linearly express a general 8-D polarisation-entangled state:

$$\begin{aligned}\left|\psi\right\rangle = {} & \alpha_1\left|H\right\rangle\left|H\right\rangle\left|H\right\rangle + \alpha_2\left|V\right\rangle\left|H\right\rangle\left|H\right\rangle + \alpha_3\left|H\right\rangle\left|V\right\rangle\left|H\right\rangle \\ & + \alpha_4\left|H\right\rangle\left|H\right\rangle\left|V\right\rangle + \alpha_5\left|V\right\rangle\left|V\right\rangle\left|H\right\rangle + \alpha_6\left|V\right\rangle\left|H\right\rangle\left|V\right\rangle \\ & + \alpha_7\left|H\right\rangle\left|V\right\rangle\left|V\right\rangle + \alpha_8\left|V\right\rangle\left|V\right\rangle\left|V\right\rangle. \end{aligned} \tag{S.31}$$

By comparing the representation of vector beam ($|\psi\rangle = \alpha|\ell_1\rangle|H\rangle + \sqrt{1-\alpha^2}|\ell_2\rangle|V\rangle$) and Eqs. (S.24) and (S.25), the vector beams show an excellent classical picture corresponding to the quantum Bell states, because the non-separability of transverse mode and polarisation DoFs in vector beam is just corresponding to the non-separability of two polarisation states in entangled photon pair. Thus this exotic non-separability in vector beams is also called classical entanglement. However, this previous works are constrained within the 2-D Bell states. In order to transfer GHZ states into classical entanglement, we should find more DoFs beyond transverse mode and polarisation. It seems difficult to find more DoFs because the transverse mode and polarisation are the basic elements constituting a common laser beam. In contrast to the common beams, the geometric beams in SU(2) coherent states have a coupling effect between the transverse and longitudinal modes induced by the ray-wave duality. We can elaborately modulate the intensity and polarisation of each periodic orbit to manipulate three DoFs and realize GHZ states alternatively: the first DoF is the oscillating direction, the second is the location of periodic orbits, the third is the polarisation, then the classical GHZ states yield:

$$\left|\Phi^{\pm}\right\rangle = \frac{\left|+\right\rangle\left|1\right\rangle\left|D\right\rangle \pm \left|-\right\rangle\left|2\right\rangle\left|A\right\rangle}{\sqrt{2}}, \tag{S.32}$$

$$\left|\Psi_1^{\pm}\right\rangle = \frac{\left|-\right\rangle\left|1\right\rangle\left|D\right\rangle \pm \left|+\right\rangle\left|2\right\rangle\left|A\right\rangle}{\sqrt{2}}, \tag{S.33}$$

$$\left|\Psi_2^{\pm}\right\rangle = \frac{\left|+\right\rangle\left|2\right\rangle\left|D\right\rangle \pm \left|-\right\rangle\left|1\right\rangle\left|A\right\rangle}{\sqrt{2}}, \tag{S.34}$$

$$\left|\Psi_3^{\pm}\right\rangle = \frac{\left|+\right\rangle\left|1\right\rangle\left|A\right\rangle \pm \left|-\right\rangle\left|2\right\rangle\left|D\right\rangle}{\sqrt{2}}. \tag{S.35}$$

where $|+\rangle$ and $|-\rangle$ represent the positive and negative SU(2) oscillating states, $|1\rangle$ and $|2\rangle$ are the first and second round-trip locations of periodic orbits, here we change the polarisation states from horizontal and vertical linear-polarisation states $|H\rangle$ and $|V\rangle$ into diagonal and antidiagonal linear-polarisation state $|D\rangle = \frac{1}{\sqrt{2}}(|H\rangle + |V\rangle)$ and $|A\rangle = \frac{1}{\sqrt{2}}(|H\rangle - |V\rangle)$ only because it is corresponding to our experimental description more conveniently.

D. Projected Bell states from GHZ states

Via intensity modulation by iris on I_1 or I_2 and SLM with "$\pi/2|3\pi/2$" or "$3\pi/2|\pi/2$" phase-step mask, four orthogonal vector beams corresponding to the four maximumly entangled groups $|\Phi^{\pm}\rangle$, $|\Psi_1^{\pm}\rangle$, $|\Psi_2^{\pm}\rangle$, and $|\Psi_3^{\pm}\rangle$ would be obtained. By using the polarizer, we measured the vector properties in these beams. Figure S2 shows the theoretical and experimental results of the intensity pattern of the four maximumly entangled groups before and after polarizer measure. For $|\Phi^{\pm}\rangle$ and $|\Psi_3^{\pm}\rangle$, the intensity pattern is diagonal, and polarisations are orthogonal at the corresponding orbit location, i.e. $|1\rangle|D\rangle$ with $|2\rangle|A\rangle$ and $|1\rangle|A\rangle$ with $|2\rangle|D\rangle$. For $|\Psi_1^{\pm}\rangle$ and $|\Psi_2^{\pm}\rangle$, the intensity pattern is antidiagonal, polarisation distribution are $|1\rangle|D\rangle$ with $|2\rangle|A\rangle$ and $|1\rangle|A\rangle$ with $|2\rangle|D\rangle$. So far, we cannot say we obtain the vector beams completely expressed in 8-D space because we cannot distinguish the "$\pm$" signals and get the complete tomography of the 8 GHZ states.

Hereinafter, we demonstrate how to distinguish the "$\pm$" states in the four groups of maximum entanglement. Actually, the "+" and "$-$" states are corresponding to the classical orbits with phase differences of 0 and π. The phase difference of two classical light can be revealed by observation of interference fringes. In order to observe interference fringes of orbits, we should project the vector beams into a certain polarisation for producing coherence. According to the properties of GHZ states, a GHZ state will be reduced into a Bell state after polarisation projection. For classical GHZ states, different Bell states will also be obtained after polarisation projection, yielded by:

$$\begin{aligned}\left|\Phi^{\pm}\right\rangle &= \frac{1}{\sqrt{2}}\left(\left|+\right\rangle\left|1\right\rangle\frac{\left|H\right\rangle+\left|V\right\rangle}{\sqrt{2}} \pm \left|-\right\rangle\left|2\right\rangle\frac{\left|H\right\rangle-\left|V\right\rangle}{\sqrt{2}}\right)\\ &= \frac{\left|+\right\rangle\left|1\right\rangle \pm \left|-\right\rangle\left|2\right\rangle}{2}\left|H\right\rangle + \frac{\left|+\right\rangle\left|1\right\rangle \mp \left|-\right\rangle\left|2\right\rangle}{2}\left|V\right\rangle\\ &= \frac{\left|\psi^{\pm}\right\rangle\left|H\right\rangle + \left|\psi^{\mp}\right\rangle\left|V\right\rangle}{\sqrt{2}},\end{aligned} \tag{S.36}$$

$$\begin{aligned}\left|\Psi_1^{\pm}\right\rangle &= \frac{1}{\sqrt{2}}\left(\left|-\right\rangle\left|1\right\rangle\frac{\left|H\right\rangle+\left|V\right\rangle}{\sqrt{2}} \pm \left|+\right\rangle\left|2\right\rangle\frac{\left|H\right\rangle-\left|V\right\rangle}{\sqrt{2}}\right)\\ &= \frac{\left|-\right\rangle\left|1\right\rangle \pm \left|+\right\rangle\left|2\right\rangle}{2}\left|H\right\rangle + \frac{\left|-\right\rangle\left|1\right\rangle \mp \left|+\right\rangle\left|2\right\rangle}{2}\left|V\right\rangle\\ &= \frac{\pm\left|\phi^{\pm}\right\rangle\left|H\right\rangle \mp \left|\phi^{\mp}\right\rangle\left|V\right\rangle}{\sqrt{2}},\end{aligned} \tag{S.37}$$

$$\begin{aligned}\left|\Psi_2^{\pm}\right\rangle &= \frac{1}{\sqrt{2}}\left(\left|+\right\rangle\left|2\right\rangle\frac{\left|H\right\rangle+\left|V\right\rangle}{\sqrt{2}} \pm \left|-\right\rangle\left|1\right\rangle\frac{\left|H\right\rangle-\left|V\right\rangle}{\sqrt{2}}\right)\\ &= \frac{\left|+\right\rangle\left|2\right\rangle \pm \left|-\right\rangle\left|1\right\rangle}{2}\left|H\right\rangle + \frac{\left|+\right\rangle\left|2\right\rangle \mp \left|-\right\rangle\left|1\right\rangle}{2}\left|V\right\rangle\\ &= \frac{\left|\phi^{\pm}\right\rangle\left|H\right\rangle + \left|\phi^{\mp}\right\rangle\left|V\right\rangle}{\sqrt{2}},\end{aligned} \tag{S.38}$$

$$\begin{aligned}\left|\Psi_3^{\pm}\right\rangle &= \frac{1}{\sqrt{2}}\left(\left|+\right\rangle\left|1\right\rangle\frac{\left|H\right\rangle-\left|V\right\rangle}{\sqrt{2}} \pm \left|-\right\rangle\left|2\right\rangle\frac{\left|H\right\rangle+\left|V\right\rangle}{\sqrt{2}}\right)\\ &= \frac{\left|+\right\rangle\left|1\right\rangle \pm \left|-\right\rangle\left|2\right\rangle}{2}\left|H\right\rangle - \frac{\left|+\right\rangle\left|1\right\rangle \mp \left|-\right\rangle\left|2\right\rangle}{2}\left|V\right\rangle\\ &= \frac{\left|\psi^{\pm}\right\rangle\left|H\right\rangle - \left|\psi^{\mp}\right\rangle\left|V\right\rangle}{\sqrt{2}},\end{aligned} \tag{S.39}$$

where the classical Bell states should be defined as:

$$|\phi^{\pm}\rangle = \frac{|+\rangle\,|2\rangle \pm |-\rangle\,|1\rangle}{\sqrt{2}}, \tag{S.40}$$

$$|\psi^{\pm}\rangle = \frac{|+\rangle\,|1\rangle \pm |-\rangle\,|2\rangle}{\sqrt{2}}. \tag{S.41}$$

After projection onto $|H\rangle$ and $|V\rangle$ states, $|\Phi^{\pm}\rangle$ or $|\Psi_3^{\pm}\rangle$ state would be reduced to $|\psi^{\pm}\rangle$ and $|\psi^{\mp}\rangle$ states, $|\Psi_1^{\pm}\rangle$ or $|\Psi_2^{\pm}\rangle$ to $|\phi^{\pm}\rangle$ and $|\phi^{\mp}\rangle$. The "+" and "−" in Bell states can be distinguished by the complementary interferometric fringes of the corresponding phase difference of 0 and π between two SU(2) orbits. For measuring $|\Phi^{\pm}\rangle$ and $|\Psi_3^{\pm}\rangle$, the CCD camera should be located at $z = -z_R$ position where $|-1\rangle$ and $|+2\rangle$ orbits are overlapped. For $|\Psi_1^{\pm}\rangle$ and $|\Psi_2^{\pm}\rangle$, the CCD camera should be located at $z = z_R$ position where $|+1\rangle$ and $|-2\rangle$ orbits are overlapped. Without polarisation projection, the pattern shows no fringes because the light on the corresponding two orbits are incoherent. After projection on $|H\rangle$ or $|V\rangle$ states, different interference fringes would be observed for different reduced Bell states. For the group $|\Phi^{\pm}\rangle$, the "±" cannot be distinguished by the intensity patterns. However if we project the polarisation onto $|H\rangle$ state to observe the pattern of $\langle H|\Phi^{\pm}\rangle$, pattern of original state $|\Phi^{\pm}\rangle$ will be reduced into Bell states $|\psi^{\pm}\rangle$ and two different patterns of complementary fringes will be observed, center-bright fringes for $|\psi^{+}\rangle$ (the original state should be $|\Phi^{+}\rangle$) and center-dark fringes for $|\psi^{-}\rangle$ (the original state should be $|\Phi^{-}\rangle$). We can also use projected state $\langle V|\Phi^{\pm}\rangle$ to distinguish the "±" that $\langle V|\Phi^{+}\rangle$ should be center-dark fringes corresponding to Bell state $|\psi^{-}\rangle$, and $\langle V|\Phi^{-}\rangle$ should be center-bright fringes corresponding to Bell state $|\psi^{+}\rangle$. In experiment, we can use a BK7 thin plate to cover one of the two orbits and rotate slightly to control the phase difference between them to control a phase difference of π, switching from "+" to "−" state. Other GHZ states can be generated by the similar way fulfilling a completed set in 8-D Hilbert space.

E. Generating general high-dimensional classical entanglement from a laser

The general SU(2) vector beams with general high-dimensional entanglement can be directly generated in a cavity. In other words, a degenerate cavity with off-axis displacement should commonly generate the general SU(2) vector beams while normal SU(2) scalar beams are just specific cases. Actually, considering the anisotropism in gain medium induced by crystal cutting geometry and nonuniform thermal effect by asymmetric pumping, the geometric beams can undergo complex amplitude and polarisation modulations in cavity and be output as vector fields. In our experiment, we used a c-cut $Nd{:}YVO_4$ as gain medium to realize intracavity complex amplitude and polarisation modulations. $Nd{:}YVO_4$ is a positive uniaxial crystal with anisotropic refractive indices and stimulated absorption and emission cross-sections. The detailed schematic for arranging the crystal in our experiment is depicted in Fig. S4. In c-cut $Nd{:}YVO_4$, the principal c-axis is located on z-axis and other two a-axes on the (x, y) transverse plane. For a light beam with normal incidence, there is no birefrigent effect; for a beam with a incident angle $\theta_{\rm in}$, there is a birefrigent modulation where the vertical linear polarized component undergoes an ordinary refractive index $n_{\rm o}$ and angle $\theta_{\rm o}$ while the horizontal linear polarized component undergoes an effective refractive index $n_{\rm eff}$ and angle $\theta_{\rm eff}$ involved in the ordinary and extraordinary refractive indices $n_{\rm o}$ and $n_{\rm e}$:

$$n_{\rm eff} = \frac{n_{\rm o} n_{\rm e}}{\sqrt{n_{\rm e}^2 \cos^2\theta_{\rm in} + n_{\rm o}^2 \sin^2\theta_{\rm in}}}. \tag{S.42}$$

The difference of the two refractive indices leads to the phase retardation between the orthogonal polarized components, which can be represented as

$$\Delta = \frac{2\pi d}{\lambda_0}\left(\frac{n_{\rm eff}}{\cos\theta_{\rm eff}} - \frac{n_{\rm o}}{\cos\theta_{\rm o}}\right), \tag{S.43}$$

where d is the thickness of the crystal. According to Eq. (S.43), when the laser crystal is given (d and λ_0 is determined), the polarisation control of geometric mode can be experimentally realized by two methods of: (1) modulating the refractive indices; (2) modulating the incidence angle, i.e. the included angle of classical orbits of geometric mode. The method-(1) can be realized by control of pump power, and the method-(2) can be realized by control of off-axis displacement.

Modulation by pump power. It has been proved that the temperature-dependent thermal effects in $Nd{:}YVO_4$ solid-state laser is highly related to the pump power, while the refractive indices, stimulated absorption and emission cross-sections are all related to the thermal effect [12–14]. Thus the different powers corresponding to different thermal effects and then to different refractive indices. In SU(2) geometric mode, the pump spot is off-axis, thus nonuniform thermal effect is nonuniform and the polarisation modulation is also nonuniform, resulting into the output of SU(2) vector beams.

Modulation by off-axis displacement. According to the ray-wave duality, the SU(2) geometric modes with different orders have different incident angles for various orbits and the ray trajectory is coupled with the pumping spot. Therefore, the control of pumping spot is related to the included angles of incidence rays in the classical trajectory. The different off-axis displacements corresponding to different transverse-orders of geometric mode result into different incidence angles. Note that the nonuniform thermal distribution and transverse-order comprehensively impact on the polarisation of geometric mode.

Therefore, there should be asymmetrical complex amplitude and polarisation modulations by general pumping control. For instance, Fig. S5(a) shows an experimental result of an SU(2) geometric mode in state

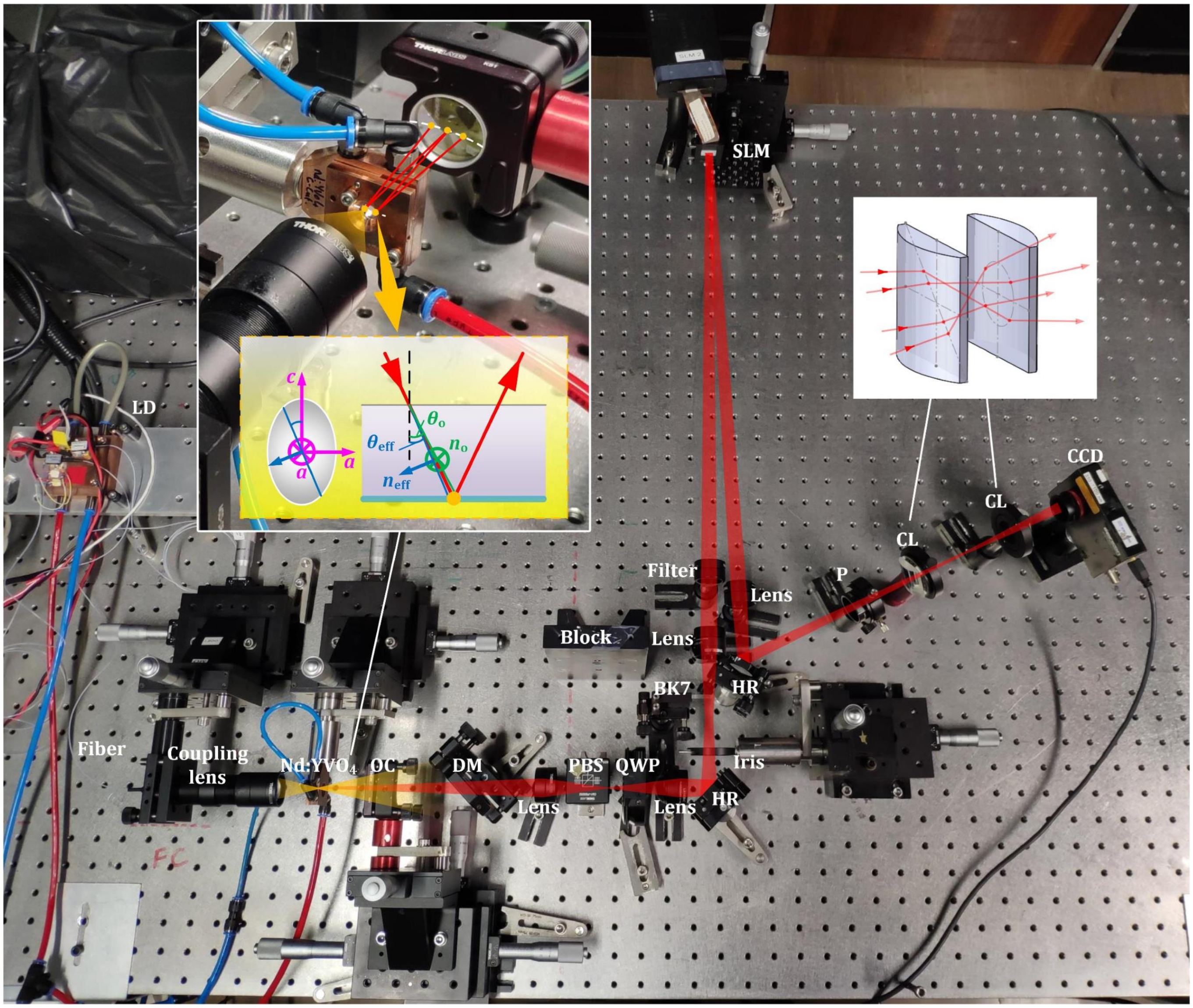

FIG. S4. **Experimental setup.** The actual picture is in accord with the experimental schematic of Fig. 4 in main article, where one insert (left) shows the details of the arrangement of c-cut Nd:YVO$_4$, another insert (right) shows the two cylindrical lenses playing as astigmatic mode converter with the ray-represent of the transformation from a planar geometric mode to a vortex geometric mode. The trajectory of SU(2) geometric mode is depicted in the cavity. The index ellipsoide is depicted for determining the effective refraction index and angle of a geometric beam in crystal. OC: output coupler; DM: dichroic mirror; PBS: polarisation splitting prism; QWP: quarter-wave plate; HR, high-reflective mirror; SLM, spatial light modulator; P: polarizer; CL: cylindrical lens; CCD: charge-coupled device.

$|\Omega = 1/4\rangle|\phi = \pi\rangle$, where different orbits show different intensities and polarisations. For each orbit, the left- and right-handed circular polarized components are measured by a polarisation grating, which uses geometric phase to diffract light into two beams in the $+1$ and -1 orders such that the two output beams have opposite circular polarisations, and the horizontal and vertical linear polarized components are measured by a rotating polarizer. Through the polarisation components measurement, we can evaluate the actual polarisations of light on various orbits. The experimental results show that the SU(2) geometric beam is a general vector state, i.e. the lights on different orbits have different polarisation and intensity.

The actual vector property can also be revealed by the interference fringes emerged at $z = 0$ and $z = \pm z_R$ position. By adjusting the pumping power and displacement, we can modulate the vector field in the output general SU(2) vector beam, modulating the polarisations on $|+1\rangle$ and $|-2\rangle$ orbits from coherent to orthogonal. The evolution of the transverse pattern at $z = z_R$ shows the coherence between $|+1\rangle$ and $|-2\rangle$ orbits changing from weak to strong with the interference fringes from vague to clear, unraveling the polarisations on the two orbits changing into orthogonal states. This experimentally observed evolution is shown in Fig. S5(b).

In summary, using off-axis pumping in a frequency-

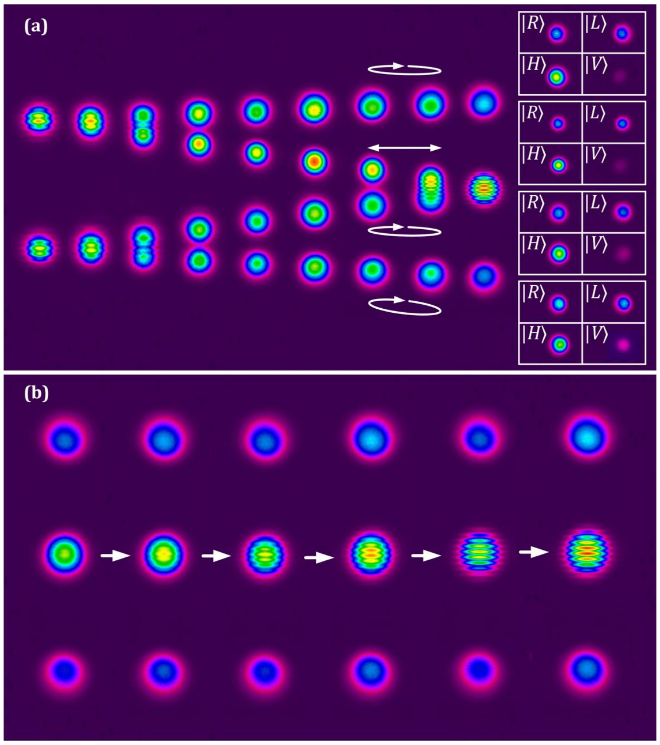

FIG. S5. **Experimental generation of general SU(2) vector beams.** (a) The experimental measure of transverse patterns versus the propagation distance of an SU(2) geometric mode in state $|\Omega = 1/4\rangle|\phi = \pi\rangle$, where different orbits show different intensities and polarisations. (b) The experimentally observed evolution of the transverse pattern at $z = z_R$ plane with the adjustment of the pumping power and displacement.

degenerate cavity with c-cut $Nd{:}YVO_4$ as gain medium, general SU(2) vector beams with high-dimensional entanglement property can be generated.

F. Experimental setup details

The main setup is depicted Fig. S4. The frontend is a laser oscillator with cavity precisely adjusted into frequency-degenerate state $|\Omega = 1/4\rangle$ and phase state $|\phi = \pi\rangle$. Through controlling the off-axis pumping, a ray-like SU(2) coherent state wave-packet can be generated and coupled with a W-shape classical geometric trajectory. The positive and negative oscillating states $|+\rangle$ and $|-\rangle$ share the overlapped trajectory. Here the signal is defined by the projection of photon propagating direction on x-axis. There are two round-trip orbits bouncing at flat mirror, i.e. the two V-shape orbits, noted as $|1\rangle$ and $|2\rangle$, for the coherent state $|\Omega = 1/4\rangle|\phi = \pi\rangle$ [15]. The intracavity geometric trajectory is constituted by four periodic oscillating orbits $|+1\rangle$, $|+2\rangle$, $|-1\rangle$, and $|-2\rangle$, each of them contributes a ray-like orbit in the actual output SU(2) geometric mode. The planar geometric mode can be astigmatically converted into vortex geometric mode with OAM where the corresponding states $|\pm\rangle$, $|1\rangle$ and $|2\rangle$ are located at a hyperbolic ruled surface with orthogonal SU(2) coordinates as illustrated in the introduction of concept in the main text.

A 808 nm fiber-coupled laser diode (LD) (FOCUSLIGHT, FL-FCSE08-7-808-200) was used as the pump source. With a telescope system with magnification about 1:1 constituted by two identical anti-reflective (AR) coated lenses (focal length $F = 25$ mm), the pump light was focused into a c-cut $Nd{:}YVO_4$ slice-like crystal with dopant of 0.5 at.% and thickness of 5 mm, which was wrapped in a copper heat sink and conductively water cooled at 18°C. The outside surface of crystal was coated AR at 808 nm and high-reflective (HR) at 1064 nm and the inner surface AR at 1064 nm. A plano-concave mirror was used as the output coupler, where the radius of curvature is 100 mm and the transmittance is 10% at 1064 nm for inner surface and AR for outside surface.

The laser was firstly passed through a dichroic mirror (DM, 45° incidence, HR at pump light and AR at laser) for filtering residual pumping light. After lens focusing and passing through a polarisation splitting prism (PBS) and quarter-wave plate (QWP), we can control the SU(2) geometric beam as right-hand circular polarisation state. Using a high-reflective mirror and lens, we can illuminate the SU(2) geometric beam on a spatial light modulator (SLM) with beam waist location overlapped with the phase mask and incidence angle less than 5°. A filter was used before the illumination on SLM for avoiding over-power damage. The phase modulation is only sensitive to horizontal linear polarisation component, thus it can be used to modulate polarisation and generate SU(2) structured vector beams by phase mask design. For a input light with right-hand circular polarisation, if the phase mask plays a $\pi/2$ constant phase, the output will be diagonal linear polarisation, and if $3\pi/2$, anti-diagonal linear polarisation. Here we equally divided the phase mask into two parts, one for modulating the polarisation on $|1\rangle$ and another for $|2\rangle$. When the SLM add divided phase "$\pi/2|3\pi/2$" on the mask, $|1\rangle|D\rangle$ and $|2\rangle|A\rangle$ states can be produced, and when "$3\pi/2|\pi/2$" on phase mask, $|1\rangle|A\rangle$ and $|2\rangle|D\rangle$ states are produced.

For introducing more structure control, we used an iris to make on-demand intensity modulation. Due to the special spatial structure of the SU(2) geometric beam, different effects would be introduced for different location of iris application. When the iris with a proper aperture size was applied at negative Rayleigh length position (I_1 position), the orbits $|-1\rangle$ and $|+2\rangle$ could be blocked, resulting in a diagonal intensity pattern in the corresponding vortex SU(2) geometric beam. When applied at positive Rayleigh length position (I_2 position), $|+1\rangle$ and $|-2\rangle$ could be blocked, resulting in anti-diagonal intensity pattern vortex SU(2) geometric beam. A BK7 thin plate can be placed partially at the beam waist position for adding a phase difference for $|1\rangle$ or $|2\rangle$ states. After intensity modulation, the structured light can no longer be a normal SU(2) geometric mode, we here call the new structured light as general SU(2) geometric beams and

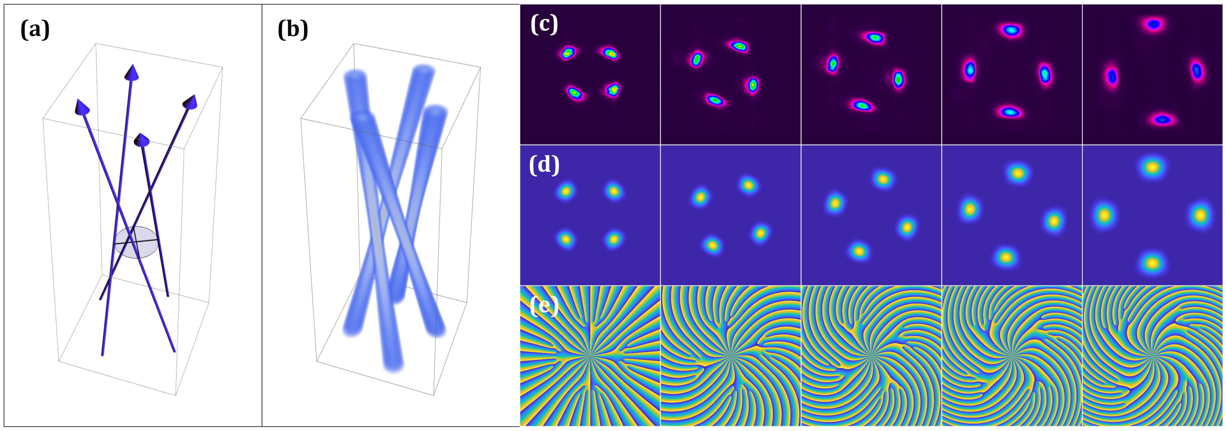

FIG. S6. **OAM measurement of SU(2) geometric vortex beam.** (a) the theoretical twisted ray trajectory and (b) the spatial intensity wave-packet of an SU(2) geometric vortex beam. (c) The experimental measured transverse patterns of an SU(2) geometric vortex beam at various propagation distances from $z = 0$ to $z = z_R$, and the corresponding theoretical simulations of (d) transverse patterns and (e) phases.

the beam after polarisation modulation as general SU(2) vector beams. Because we used a c-cut Nd:YVO_4 providing anisotropic intracavity polarisation modulation on geometric modes [16, 17], our laser output should be a general SU(2) vector beam. Hereinafter, we will demonstrate that the general SU(2) vector beams can be measured completely by 3-DoF bases in 8-D Hilbert space and a complete set of GHZ states ($|\Phi^{\pm}\rangle$, $|\Psi_1^{\pm}\rangle$, $|\Psi_2^{\pm}\rangle$, and $|\Psi_3^{\pm}\rangle$) can be experimentally generated and controlled.

After the SLM modulation, the tomography measure system was designed. A astigmatic mode convertor (AMC) including two 45°-inclined AR-coated cylindrical lenses was used to convert a planar general SU(2) geometric beam into vortex beam with disjoint geometric orbits. After that, a charge coupled device (CCD) camera was used to identify the diagonal and anti-diagonal intensity patterns. A rotatable polarizer should be inset before the CCD to measure the vector properties and identify the diagonal and anti-diagonal linear polarisation states for each orbit. The combination of this measure results in the tomography of for vector beams corresponding to the four maximumly entangled groups of $|\Phi^{\pm}\rangle$, $|\Psi_1^{\pm}\rangle$, $|\Psi_2^{\pm}\rangle$, and $|\Psi_3^{\pm}\rangle$. The "±" states are revealed by the phase difference on a certain orbit, which can be identified by the interference fringes of corresponding two orbits. For the SU(2) coherent state $|\Omega = 1/4\rangle|\phi = \pi\rangle$, the interference of $|+\rangle$ and $|-\rangle$ orbits occurred at the beam waist, and the interference of $|1\rangle$ and $|2\rangle$ orbits occurred at the Rayleigh length z_R away from the beam waist. For identifying "±" in GHZ states, the measure of the interference fringes is required. Before the measure of the interference fringes, a polarizer was used to project the polarisation on a unified $|H\rangle$ or $|V\rangle$ state, in order to provide coherence, just corresponding to the process of Bell state projection. When the CCD was located at $z = -z_R$, the interference for $|-1\rangle$ and $|+2\rangle$ can be detected, and when $z = -z_R$, the interference for $|+1\rangle$ and $|-2\rangle$ can be detected.

Without the SLM modulation, we can detect the scalar SU(2) geometric vortex beam after the astigmatism lens converter of the original state, where the lights on various ray states are all observable. Figures S6(a) and S6(a) show the theoretically predicted spatial twisted ray trajectory and the corresponding spatial wave-packet, and the experimentally measured results are in Fig. S6(c), where the four-lobe square-shaped pattern angularly rotates along the z-axis with an increase in propagation distance, which agree well with the simulated results [Fig. S6(d)], we also can used the simulated phase [Fig. S6(e)] to reveal the actual OAM and topological charges of the beam.

G. Quantitative measurement of OAM

From the above information, the phase distribution of an SU(2) geometric vortex beam has a spiral phase structure a characteristic of light fields carrying OAM. The spiral phase pattern resides in the core region with darkness. It is important to provide an experimental measurement to verify such a phase pattern and quantitatively measure the value of its topological charge, for clear identification of the OAM state.

Additionally, the methods to detect the vortex topological phase surrounding the darkness were previously studied. A direct method is to interfere the generated mode with a reference coherent planar [8]. The vortex phase can then be verified by comparing the theoretical and experimental interference fringes. However, this method is difficult to precisely determine the topological charge, because brightness regions are not continuous in the SU(2) geometric vortex therefore making it is difficult to count all the fringes with high visibility. A more precise method has been proposed [9], which involves using a truncated aperture and observing the resulting far-field diffraction pattern (optical lattices pattern) to quantitatively measure the OAM topological charge in an SU(2)

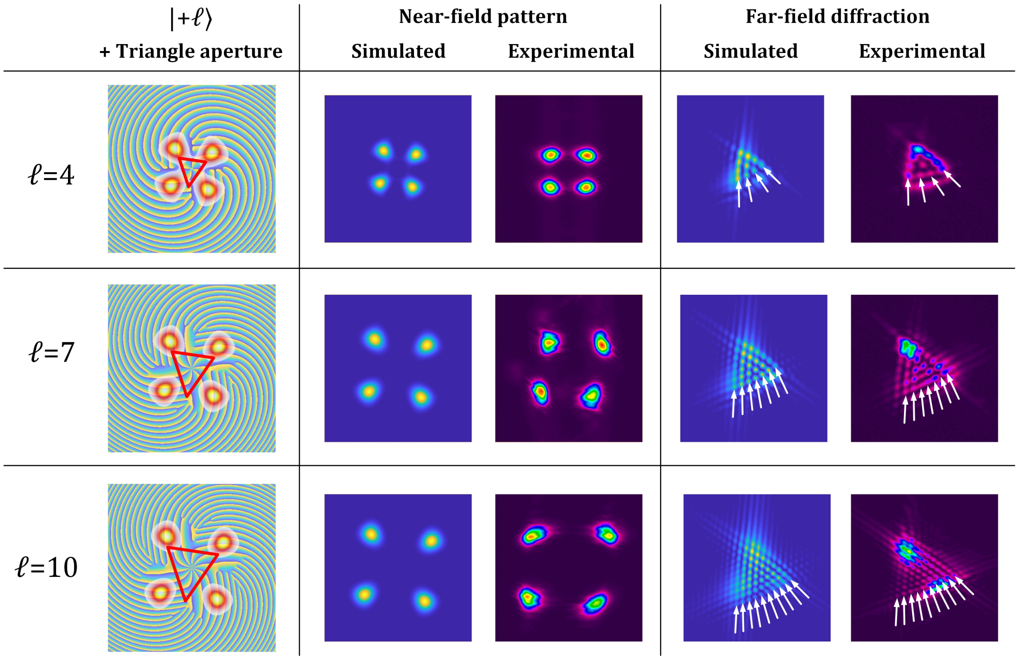

FIG. S7. **Quantitative measurement of OAM.** The left column shows three SU(2) geometric beams (intensity and phase) with OAM charges $\ell = 4, 7, 10$, respectively. The location of the triangle aperture (marked by red triangle) for each case is illustrated in the first column. The middle column shows the theoretical and experimental near-field patterns before passing through triangle apertures for the three cases. The last column shows the far-field diffraction patterns after truncation of corresponding triangle apertures for the three cases, which show optical lattice pattern. Counting the spots in the far-field lattice structure, reveals the corresponding values of OAM topological charges, as marked by the white arrows.

geometric vortex beam. This is both available to measure the center dark OAM topological charge and the partial OAM of the sub-beam located topological charge.

The method is demonstrated as follows. A triangle truncated aperture is applied to the center of an SU(2) geometric vortex beam. The size of the aperture is set to cut the inner edge of the brightness region, the smaller or larger of which would not impact on the measured results. We can then observe the corresponding triangle optical lattice pattern at the far-field diffraction plane. Theoretically, the number of spots in the optical lattices is directly related to the OAM of the measured beam. The topological charge can be then be measured by counting the spots in the optical lattices pattern. We show the results of such measure processes for three SU(2) geometric beams at OAM states $|\ell\rangle$ with different values of topological charges, $\ell = 4, 7, 10$, respectively, in Fig. S7. This method is both available to measure the center dark OAM topological charge and the partial OAM of the sub-beam located topological charge, just by adjusting the location of the triangle aperture the corresponding dark regions (see more details in Ref. [9]).

H. Towards higher-dimensional classically entangled state

Hereto, we can already generate a complete set of GHZ states from a laser, sharing the same form of three-photon quantum entanglement. The general SU(2) modes also have potential to be used to generate even higher-dimensional entanglement states with the form of N-photon ($N > 3$) quantum entanglement. To this end, we should find more DoFs to extend the dimension in classical entanglement such as OAM. As shown in Fig. S8(a-d), two SU(2) modes with opposite OAM can be superposed together fulfilling a complete oscillating trajectory in a cavity, which can also be experimentally realized [18]. After inhomogeneous intensity and polarisation modulation, this kind of general SU(2) modes should be represented in 16-D Hilbert space like the 4-photon quantum entanglement:

$$\begin{aligned}|\psi\rangle = {} & \alpha_1 |{+\ell}\rangle |+\rangle |1\rangle |H\rangle + \alpha_2 |{-\ell}\rangle |+\rangle |1\rangle |H\rangle \\ & + \alpha_3 |{+\ell}\rangle |+\rangle |1\rangle |V\rangle + \alpha_4 |{-\ell}\rangle |+\rangle |1\rangle |V\rangle \\ & + \alpha_5 |{+\ell}\rangle |-\rangle |1\rangle |H\rangle + \alpha_6 |{-\ell}\rangle |-\rangle |1\rangle |H\rangle \\ & + \alpha_7 |{+\ell}\rangle |-\rangle |1\rangle |V\rangle + \alpha_8 |{-\ell}\rangle |-\rangle |1\rangle |V\rangle \\ & + \alpha_9 |{+\ell}\rangle |+\rangle |2\rangle |H\rangle + \alpha_{10} |{-\ell}\rangle |+\rangle |2\rangle |H\rangle \\ & + \alpha_{11} |{+\ell}\rangle |+\rangle |2\rangle |V\rangle + \alpha_{12} |{-\ell}\rangle |+\rangle |2\rangle |V\rangle \\ & + \alpha_{13} |{+\ell}\rangle |-\rangle |2\rangle |H\rangle + \alpha_{14} |{-\ell}\rangle |-\rangle |2\rangle |H\rangle \\ & + \alpha_{15} |{+\ell}\rangle |-\rangle |2\rangle |V\rangle + \alpha_{16} |{-\ell}\rangle |-\rangle |2\rangle |V\rangle ,\end{aligned} \tag{S.44}$$

with 16 GHZ states, as the eigenstates of 4-partite 16-dimensional space and the 8 maximumly entangled group, as given by (just show the first maximumly en-

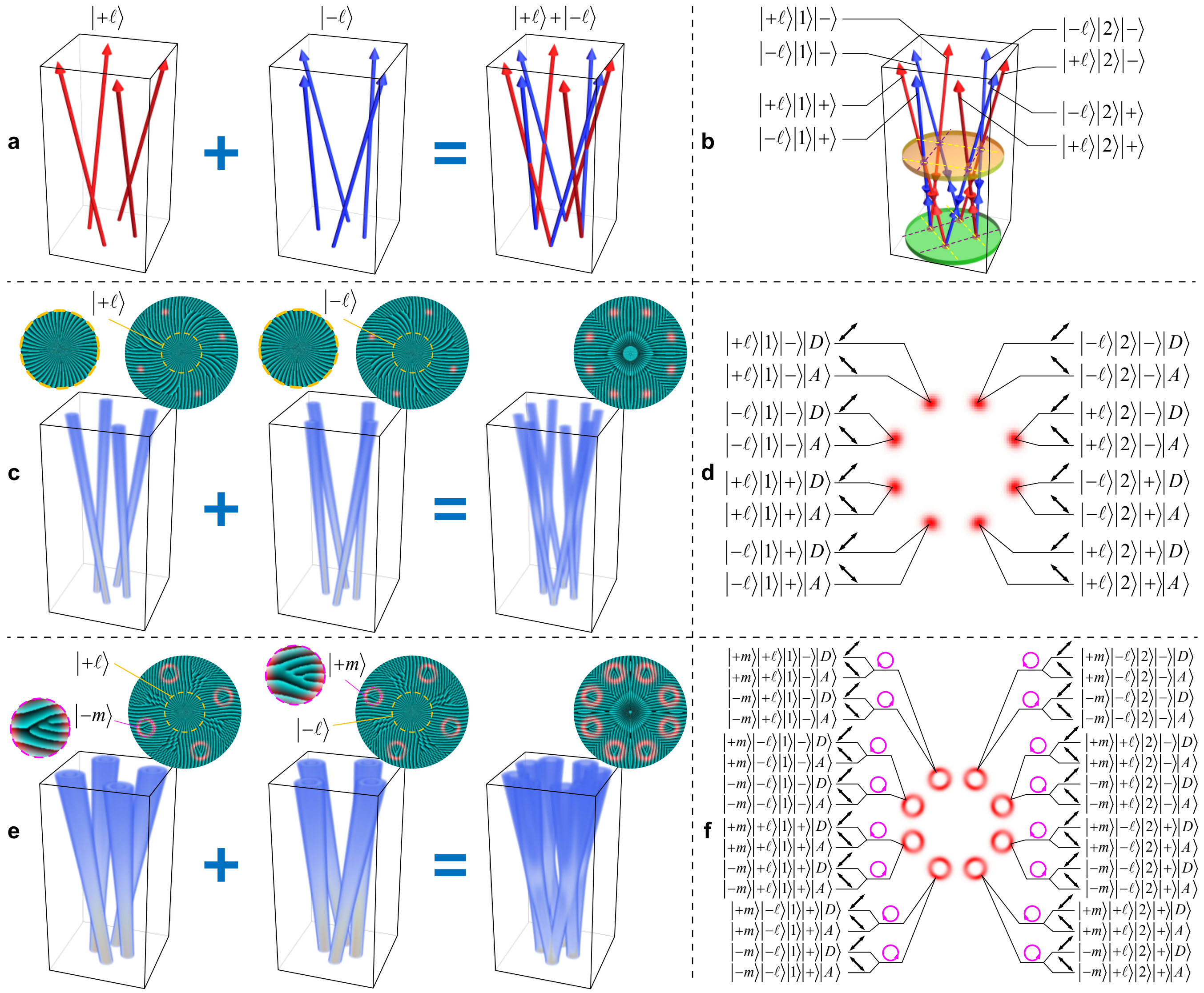

FIG. S8. **Towards higher-dimensional classical entanglement.** Spatial SU(2) geometric beams can carry OAM and the OAM can be a DoF manifesting additional dimensions in classical entanglement. (a) A superposed trajectory on SU(2) state $|\Omega = 1/4\rangle$ including the positive-OAM and negative-OAM decomposed SU(2) trajectories, (b) which can exactly fulfill a complete SU(2) oscillation in a degenerate cavity. (c) Based on the ray-wave duality, the corresponding geometric mode can be represented by the superposition of a positive-OAM and a negative-OAM vortex SU(2) geometric beams (the topological phase manifesting the OAM and intensity wave-packet are shown in the inserts). This generalized beam can be expressed into 16-dimensional state with 4 DoFs and 16 eigenstates noted in figure (d). (e) An exotic multi-vortex SU(2)-structured mode is obtained by the superposition of two multi-vortex SU(2) modes with opposite main-OAMs, and the sub-OAM carried by each sub-ray mode can also play as a new DoF. This generalized beam can be expressed into 32-dimensional state with 5 DoFs and 32 eigenstates noted in figure (f).

tangled group here):

$$\left|\Phi^{\pm}\right\rangle = \frac{|+\ell\rangle\,|+\rangle\,|1\rangle\,|D\rangle \pm |-\ell\rangle\,|-\rangle\,|2\rangle\,|A\rangle}{\sqrt{2}}. \tag{S.45}$$

For exploring more DoFs to extend the dimension in classical entanglement, we can utilize multi-LG SU(2) geometric beams [19, 20], where LG beams replace the Gaussian beams along SU(2) orbits in a geometric mode based on ray-wave duality. Thus, there is a main OAM state $|+\ell\rangle$ along the propagation axis, but also OAM $|+m\rangle$ along an SU(2) orbit. The sub-OAM $|+m\rangle$ has potential to be a DoF to reach higher-dimensional entanglement. As shown in Fig. S8(e,f), exotic beams can be obtained by the superposition of two multi-LG SU(2) geometric beams with opposite main OAM, realizing the 32-D entangled stated with 5-partite 16-group GHZ states as eigenstates (just show the first maximumly entangled group here):

$$\left|\Phi^{\pm}\right\rangle = \frac{|+m\rangle\,|+\ell\rangle\,|+\rangle\,|1\rangle\,|D\rangle \pm |-m\rangle\,|-\ell\rangle\,|-\rangle\,|2\rangle\,|A\rangle}{\sqrt{2}}. \tag{S.46}$$

Besides finding more DoFs, increasing the number of orbits can also extend the dimension. The above demonstrations are all at degenerate state $|\Omega = 1/4\rangle$. If we can control higher-order degenerate state $|\Omega = P/Q\rangle$ (P and Q are co-prime integers, Q is even), the general SU(2) modes would be extended into $4Q$-dimensional space:

$$|\psi\rangle = \sum_{i=\pm\ell}\sum_{j=\pm}\sum_{k=1}^{Q/2} |i\rangle\,|j\rangle\,|k\rangle\,(\alpha_{ijk}\,|H\rangle + \beta_{ijk}\,|V\rangle). \tag{S.47}$$

Involving the sub-OAM in multi-LG SU(2) geometric beams, that can be further extended into $8Q$-dimensional space:

$$|\psi\rangle = \sum_{h=\pm m}\sum_{i=\pm\ell}\sum_{j=\pm}\sum_{k=1}^{Q/2} |h\rangle\,|i\rangle\,|j\rangle\,|k\rangle \times (\alpha_{hijk}\,|H\rangle + \beta_{hijk}\,|V\rangle)\,. \tag{S.48}$$

The realization of high-dimensional classical entanglement can pave the way for developing a myriad of novel applications of quantum mechanism using classical light.

For instance, we can use a superposed OAM state, as shown in the Fig. S8(a), where the superposed SU(2) trajectory includes the positive and negative twisted decomposed SU(2) trajectories. More importantly, the superposed trajectory fulfills the condition of a complete oscillation in a degenerate cavity, as illustrated in Fig. S8(b), so that it can be directly generated from the laser. Therefore, the corresponding ray-wave beam introducing OAM as a new DoF shown in Fig. S8(c), can be expressed as a 16-dimensional and 4-partite classically entangled state, with the eigenstates shown in Fig. S8(d). For exploring even more DoFs, we can replace the Gaussian beams along the SU(2) orbits in a geometric mode based on ray-wave duality. In this generalized beam, there is not only a main OAM state $|\pm\ell\rangle$ along the propagation axis, but also OAM $|\pm\rangle$ carried by the sub-vortex beams along the SU(2) ray-orbits, as shown in Fig. S8(e). This exotic SU(2) geometric beam also satisfies the conditions of an SU(2) coherent state, realizing a 32-dimensional and 5-partite state, as noted in Fig. S8(f).

[1] Y. Chen, C. Jiang, Y. Lan, and K. Huang, Physical Review A **69**, 053807 (2004).
[2] Y. Chen, S. Li, Y. Hsieh, J. Tung, H. Liang, and K. Huang, Optics Letters **44**, 2649 (2019).
[3] Y. Chen, J. Tung, P. Chiang, H. Liang, and K. Huang, Physical Review A **88**, 013827 (2013).
[4] N. Barré, M. Romanelli, M. Lebental, and M. Brunel, European Journal of Physics **38**, 034010 (2017).
[5] Y. Shen, X. Yang, D. Naidoo, X. Fu, and A. Forbes, Optica **7**, 820 (2020).
[6] Y. Shen, Z. Wan, Y. Meng, X. Fu, and M. Gong, IEEE Photonics Journal **10**, 1 (2018).
[7] V. Bužek and T. Quang, Journal of the Optical Society of America B **6**, 2447 (1989).
[8] J. Tung, H. Liang, T. Lu, K.-F. Huang, and Y.-F. Chen, Optics Express **24**, 22796 (2016).
[9] Y. Shen, X. Fu, and M. Gong, Optics Express **26**, 25545 (2018).
[10] J.-W. Pan and A. Zeilinger, Physical Review A **57**, 2208 (1998).
[11] M. Michler, K. Mattle, H. Weinfurter, and A. Zeilinger, Physical Review A **53**, R1209 (1996).
[12] Y. Shen, W. Zhang, M. Gong, Y. Meng, Y. Wang, and X. Fu, Applied Sciences **7**, 470 (2017).
[13] Y. Shen, M. Gong, E. Ji, X. Fu, and L. Sun, Optics Communications **383**, 430 (2017).
[14] Y. Shen, M. Gong, and X. Fu, Applied Physics B **124**, 85 (2018).
[15] Y. Shen, X. Yang, X. Fu, and M. Gong, Applied Optics **57**, 9543 (2018).
[16] R. Guo, Y. Shen, Y. Meng, and M. Gong, Chinese Physics B **28**, 044204 (2019).
[17] T.-H. Lu and L. Lin, Applied Physics B **106**, 863 (2012).
[18] T.-H. Lu and C. He, Optics Express **23**, 20876 (2015).
[19] T.-H. Lu, Y. Lin, Y. Cexpresshen, and K. Huang, Applied Physics B **103**, 991 (2011).
[20] P. Tuan, Y. Hsieh, Y. Lai, K. Huang, and Y. Chen, Optics Express **26**, 20481 (2018).
